# Polysaccharides of Sporoderm-Broken Spore of *Ganoderma lucidum* Modulate Adaptive Immune Function via Gut Microbiota Regulation

**DOI:** 10.1155/2021/8842062

**Published:** 2021-03-23

**Authors:** Lu Su, Dan Li, Jiyan Su, Enqi Zhang, Shaodan Chen, Chaoqun Zheng, Ting Luo, Muxia Li, Xiaohong Chen, Guoxin Huang, Yizhen Xie, Shanshan Li

**Affiliations:** ^1^State Key Laboratory of Quality Research in Chinese Medicine, Macau University of Science and Technology, Macau, China; ^2^School of Pharmaceutical Science, Guangzhou University of Chinese Medicine, Guangzhou, Guangdong, China; ^3^Guangdong Provincial Key Laboratory of Microbial Safety and Health, State Key Laboratory of Applied Microbiology Southern China, Guangdong Institute of Microbiology, Guangdong Academy of Science, Guangzhou, Guangdong, China; ^4^Guangdong Yuewei Edible Fungi Technology Co., Ltd., Guangzhou, Guangdong, China; ^5^South Medical University Affiliated Maternal & Child Health Hospital of Foshan, Foshan, Guangdong, China; ^6^Queensland University of Technology, Kelvin Grove, QLD 4059, Australia; ^7^Guangdong Laboratory Animals Monitoring Institute, Guangdong Provincial Key Laboratory of Laboratory Animals, Guangzhou, Guangdong, China; ^8^Department of Traditional Chinese Medicine, The People's Hospital of Dongying, Dongying, Shandong, China

## Abstract

*Ganoderma lucidum* (Leyss.Fr.) Karst is one of the well-known medicinal macrofungi all over the world, and mounting researches have focused on the polysaccharides derived from the spores of *G. lucidum*. In the present study, BALB/c mice (*n* = 8–10) were administered with crude polysaccharides of *G. lucidum* spores (CPGS) and the refined polysaccharides of *G. lucidum* spores (RPGS) for 30 days to investigate their effect on the adaptive immune system. Results showed that CPGS and RPGS displayed diverse effects on the lymphocyte activity in the spleen. The splenocyte proliferation activity upon mitogen was suppressed by CPGS and RPGS, while the NK cell's tumor-killing ability was promoted by CPGS. Both CPGS and RPGS could increase the proportion of naïve T cells in thymus, but only RPGS significantly uplifted the percentage of T cells, as well as the T cell subsets, in peripheral blood, and promoted the activation by upregulating the expression of costimulatory factor CD28. Moreover, 16S sequencing results showed that the effects of CPGS and RPGS were closely related to the regulation of gut microbiota. *β*-diversity of the microbiome was evidently changed by CPGS and RPGS. The phytoestrogen/polysaccharide-metabolizing bacteria (*Adlercreutzia*, *Parabacteroides*, and *Prevotella*), and an unclassified *Desulfovibrionaceae*, were remarkably enriched by CPGS or RPGS, and functions involving carbohydrate metabolism, membrane transport, and lipid metabolism were regulated. Moreover, the enrichments of *Adlercreutzia*, *Prevotella*, and *Desulfovibrionaceae* were positively related to the immune regulation by CPGS and RPGS, while that of *Parabacteroides* displayed a negative correlation. These findings suggested a promising effect of the polysaccharide from sporoderm-broken spore of *G. lucidum* in immune regulation to promote health control.

## 1. Introduction

*Ganoderma lucidum* (Leyss.Fr.) Karst belongs to the family of *Basidiomycete*, Polyporaceae of *Ganoderma*. *G. lucidum* is popular worldwide for its health promotion activity. Owing to the multiple regulation on the immune system, *G. lucidum* has been widely applied as an alternative adjunct to conventional treatment against various kinds of cancers [1, 2], such as lung cancer, breast cancer, and liver cancer. More recently, *G. lucidum* spore have been recognized as promising medicinal part of *G. lucidum*, and it has drawn increasing attention for its versatile biological activities owing to the technology development in sporoderm-broken spores [3, 4]. The bioactive substances of *G. lucidum* spores include polysaccharides, triterpenoids, peptides, amino acids, fatty acids, and trace elements, which are responsible for the anti-inflammation and antitumor activities [5, 6].

Gut microbiota is a unique and diverse ecosystem known as the “forgotten organ” of the host [7]. Gut microbiota plays an important role in host health maintenance and disease pathogenesis. Gut microbiota benefits the host by improving immunity, pathogens control, energy harvest, metabolism, and tumor immunity [8–11]. However, dysbiosis of gut microbiota has been associated with inappropriate inflammatory responses and risk for disease [12]. In addition to the microorganism itself, the metabolites of gut microbiota are also able to regulate the host's physical function. For instance, short-chain fatty acids (SCFAs) derived from the gut microbiota would activate the cholinergic anti-inflammatory pathway [13]. Therefore, gut microbiota plays an important role in the maintenance of immune homeostasis [14, 15].

Emerging evidence has revealed that bioactive components from *G. lucidum*, as well as those from spore of *G. lucidum*, exert their activities via the regulation of gut microbiota. Chang et al. [16] showed that water extract of *G. lucidum* exerted a prebiotic effect to reduce obesity in mice by modulating the gut microbiota. Guo [17] and colleagues have found that triterpenoids derived from *G. lucidum* have the potential to ameliorate lipid metabolic disorders through targeting the specific gut microbiota. Li et al. [18] manifested that *G. lucidum* polysaccharides displayed an intestinal microbiota-dependent effect on mice with chronic pancreatitis. Su [19] and colleagues have verified that extract from sporoderm-broken spores of *G. lucidum* displayed a synergy with paclitaxel by remodeling the structure of the gut microbiota. In the present study, immunomodulatory activity of the crude polysaccharides of *G. lucidum* spores (CPGS) and that of the refined polysaccharides of *G. lucidum* spores (RPGS) were compared in normal mice, as well as the possible involvement of gut microbiota in the immunity regulation of polysaccharide of *G. lucidum* spores.

## 2. Materials and Methods

### 2.1. Preparation for Polysaccharide of the Spore of *G. lucidum*

The sporoderm-broken spores of *G. lucidum* were provided by Guangdong Yuewei Edible Fungi Technology Co. Ltd. Preparation for the crude polysaccharides of *G. lucidum* spores (CPGS) and that for the refined polysaccharides of *G. lucidum* spores (RPGS) were as described by Su et al. [20]. In brief, the sporoderm-broken spores were extracted with boiling water (15 L/kg) for 2 h, concentrated under vacuum, and subjected to 2 to 3 cycles of precipitation with anhydrous ethanol at a final ethanol percentage of 85%, and finally, the precipitate was lyophilized to obtain CPGS. Yield of CPGS was 2%.

For RPGS, the CPGS was dissolved in water and dialyzed with a 3500 Da dialysis tube (MWCO). The content in the 3500 Da dialysis tube was then dialyzed in a 100 kDa dialysis tube (MWCO). The dialysate was pooled, concentrated, and lyophilized to obtain RPGS. Yield of RPGS was 0.4%.

Sugar content was determined by the phenol–sulfuric acid method using glucose as standard, and protein was quantified by the BCA protein kit (Kang Wei Shiji Co. Ltd., Beijing, China). Weight average molecular weight (Mw) was analyzed by size-exclusive high-performance liquid chromatography (HPLC). Results showed that CPGS contains 64% polysaccharides and 23% protein, and the Mw is 5∼270 kDa. RPGS contains 53% polysaccharides, but the protein was hardly detected, and the Mw is 5 kDa.

### 2.2. Animal  Experiments

Male BALB/c mice (6–8 weeks old, 18–22 g, *n* = 8–10) were provided by Guangdong Medical Laboratory Animal Center (Guangzhou, Guangdong, China). All animals were housed at 20 ± 2°C with a humidity of 50 ± 5%, using dust-free poplar chips for bedding, and lighting (12 h light–12 h dark cycle). They were fed with standard diet for rodents, and water was provided *ad libitum* during the whole experiment. The experiment was performed with the approval of the Guangdong Institute of Microbiology Laboratory Animal Ethics Committee according to the guidelines.

After 1 week of acclimation, the mice were randomly divided into four groups: normal group, lentinan group (LNT, Apeloa Pharmaceutical Co., Ltd. Z20026215), CPGS group, and RPGS group. Mice of the normal group received equal volume of distilled water, while mice of the other groups were orally administrated with the corresponding drug once daily for 30 days. According to some previous studies [4, 21, 22], the dosage of LNT, CPGS, and RPGS was 200 mg/kg, 100 mg/kg, and 100 mg/kg, respectively.

**Figure 1:**
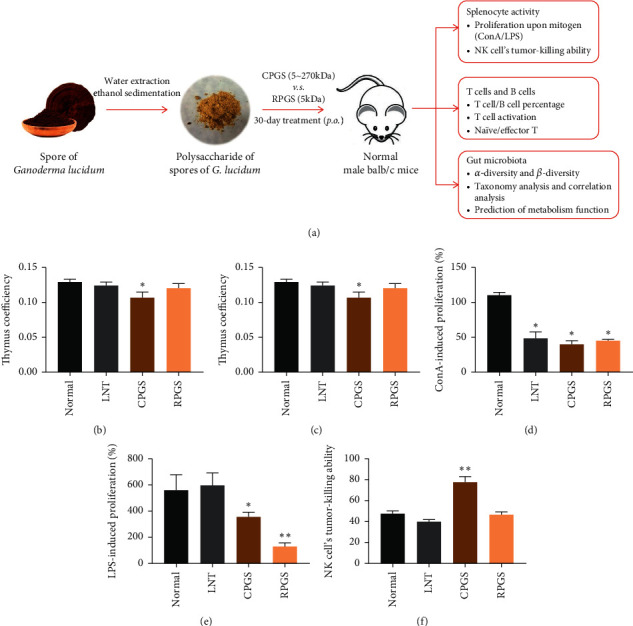
Effect of CPGS and RPGS on organ coefficient and lymphocyte activity in spleen. (a) Schematic diagram of experimental design. (b) Thymus coefficient. (c) Spleen coefficient. (d) ConA-induced Proliferation (%). (e) LPS-induced Proliferation (%). (f) NK cell's tumor-killing activity (%). Values were represented as means ± SD (*n* = 8–10).  ^*∗*^*p* < 0.05 and  ^*∗∗*^*p* < 0.01.

On the 31st day, peripheral blood was collected from the orbital vein plexus with an EDTA-Li anticoagulant tube, and the feces were collected. Then, the animals were sacrificed by cervical dislocation. Spleen and thymus were harvested, weighed, and then stored according to different purposes immediately. The organ coefficient was calculated as follows: organ coefficient (%) = organ weight/body weight × 100%. Schematic diagram of the experimental design is shown in Figure 1(a).

### 2.3. Splenocytes Proliferation Assay

The spleen was dissected under sterile conditions, cut into pieces, and ground gently in precooled PBS. The homogenates were filtered through a 200-mesh cell strainer and centrifuged at 250×*g* for 5 min. The obtained cells were resuspended with 1 mL of homemade ACK (8290 mg ammonium chloride, 1000 mg potassium bicarbonate, and 37 mg EDTA were dissolved in 1 L distilled water, pH 7.21∼7.23) for 5 min to lyse erythrocytes, and then the splenocytes were washed with precooled PBS for 3 times. Splenocytes were resuspended in RPMI-1640 medium (Hyclone, Logan, USA) containing 10% fetal bovine serum (Gibco, NY, USA) and 1% penicillin/streptomycin (Gibco, NY, USA), cultured in incubators at 37°C under an atmosphere of 5% CO_2_ for assays.

The splenocytes were seeded at a concentration of 6 × 10^5^ cells/well (100 *μ*L/well) in 96-well culture plates (ninefold wells in each group). The mitogen response assay was performed by coculture of splenocytes with concanavalin A (ConA, 6 *μ*g/mL, Sigma, USA) or lipopolysaccharide (LPS, 10 *μ*g/mL, Sigma, USA) and incubated for 72 h at 37°C. At the same time, blank wells were set, which only contains 200 *μ*L of 1% penicillin/streptomycin RPMI-1640 basic medium. The cultures were treated with 20 *μ*L/well of Cell Titer 96®AQueous MTS Reagent (Promega, Wisconsin, USA) for 4 h. Absorbances were measured at 490 nm using a Fluorescence Microplate Reader (Bio-Tek, Winooski, USA). A proliferation index was calculated by the following formula: Relative activity (%) = (polysaccharide group − blank well)/(normal group − blank well) × 100%.

### 2.4. Splenic Natural Killer (NK) Cell Activity

Spleen effector cells (2 × 10^6^ cells/well, 100 *μ*L/well) and YAC-1 cells (4 × 10^4^ cells/well, 100 *μ*L/well) were seeded into 96-well culture plates and coincubated for 4 h at 37°C. Wells with YAC-1 cells alone were set as spontaneous release group, and wells with YAC-1 cells incubated with 1% NP40 were set as maximum release group. The plates were centrifuged (1000×*g*, 5 min), and 0.1 mL of the supernatant was aspirated into a new 96-well culture plate. The supernatant was incubated with 0.1 mL of lactate dehydrogenase (LDH) reaction buffer (Tris-HCl buffer contains 280 mg sodium lactate, 17 mg nitrotetrazolium chloride, 4 mg phenazine dimethyl sulfate, 40 mg oxidized coenzyme I, pH 8.2) for another 10 min incubation. Then, 30 *μ*L HCL (1 mol/L) was added to each well, the LDH release activity was quantitated by measuring absorbance at 490 nm. NK cell activity was calculated by the following formula: LDH release activity (%) = (experimental release − spontaneous release)/(maximum release − spontaneous release) × 100%.

### 2.5. Flow Cytometry Analysis for Peripheral Blood Mononuclear Cells (PBMCs) and Lymphocytes in Thymus

For the analysis of PBMCs, 80 *μ*L blood was incubated with 60 *μ*L 0.5% BSA/PBS containing the corresponding antibody at 4°C in dark for 30 min. 2 mL homemade ACK was added to incubate for another 10 min. Then, the samples were centrifuged at 1200×*g* for 5 min and washed with PBS for once. For analysis of lymphocytes in the thymus, the thymus was dissected and ground gently in precooled PBS. The homogenates were filtered through a 200-mesh cell strainer and centrifuged at 250×*g* for 5 min. The obtained lymphocytes were washed with PBS for twice and incubated with 60 *μ*L 0.5% BSA/PBS containing the corresponding antibody at 4°C in dark for 30 min. Finally, all samples were analyzed with a FACS Canto II cytometer equipped with Diva software (version 6.1.3).

PBMCs were stained with FITC anti-mouse CD3, PerCP anti-mouse CD4, PE anti-mouse CD8, PE-Cyanine7 anti-mouse CD28, APC anti-mouse CD34, FITC anti-mouse CD3, APC anti-mouse CD19, and PE anti-mouse CD3; while lymphocytes in thymus were stained with FITC anti-mouse CD44 and APC anti-mouse CD62 L for flow cytometry. All the above antibodies were purchased from eBioscience, Thermo Fisher Scientific (Grand Isle, NY, USA).

### 2.6. 16S rRNA Gene Sequence Analysis of Gut Microbiota in Fecal Samples

Sequencing service was provided by Personal Biotechnology Co., Ltd. (Shanghai, China), and it was performed as described by Su et al. [19] with mild modification. Briefly, total DNA from feces was extracted with OMEGA Soil DNA Kit (OMEGA, Norwalk, USA). Then, we detected the DNA extraction quality with 0.8% agarose gel, using an ultraviolet spectrophotometer quantified DNA. V3–4 region of the bacterial 16S rRNA gene were amplified by PCR with the forward primer (5′-AYTGGGYDTAAAGNG-3′) and the reverse primer (5′-TACNVGGGTATCTAATCC-3′) and then purified using AP-GX- 500 DNA Gel Extraction Kit (Axygen, Corning, USA). The library was built up with the obtained products and sequenced on a MiSeq sequencing platform (Illumina, San Diego, USA).

### 2.7. Bioinformatics Analysis

Bioinformatics analysis for the 16S amplicon sequencing data was performed on the Quantitative Insights into Microbial Ecology (QIIME) Version 1.8.0 MOTHUR (version 1.31.2) and PICRUSt (http://picrust.github.io/picrust/) [20] by R software. Bacterial operation taxonomic units (OTUs) were obtained using the uclust function in QIIME. Venn diagram, ACE, Chao, Simpson, and Shannon indexes were employed to compare *α*-diversity between groups. Principle component analysis (PCA), uniFrac distance-based principal coordinates analysis (PCoA), and analysis of similarities (ANOSIM) were applied to compare *β*-diversity. In addition to taxonomy comparison, analysis of linear discriminant analysis effect size (LEfSe) was employed to figure out the specific bacteria responsible for each group. The predicted genes and their functions were aligned to the Kyoto Encyclopedia of Genes and Genomes (KEGG) database, and differences among groups were compared with STAMP4 [20].

### 2.8. Statistics

Statistical analysis was performed with SPSS 22 (IBM Corp., NY, USA). Datasets from each experiment were subjected to a normal distribution test firstly. If in compliance with the normal distribution, the data was analyzed by Student's *t*test, and the relationship between microbes and immunity was analyzed by Pearson's correlation; otherwise, the data was compared by Mann–Whitney test, and the relationship was analyzed by Spearman's correlation. Statistical differences were considered significant at *p* < 0.05.

## 3. Results

### 3.1. CPGS and RPGS Displayed Diverse Effect on the Lymphocyte Activity in Spleen

Effects of LNT, CPGS, and RPGS on immune organs were compared by organ coefficiency. Results from Figures 1(b) and 1(c) showed that the thymus index of CPGS group was significantly lower than that of the normal group (*p* < 0.05), while spleen coefficiencies were significantly higher in CPGS group and RPGS group (*p* < 0.05).

**Figure 2:**
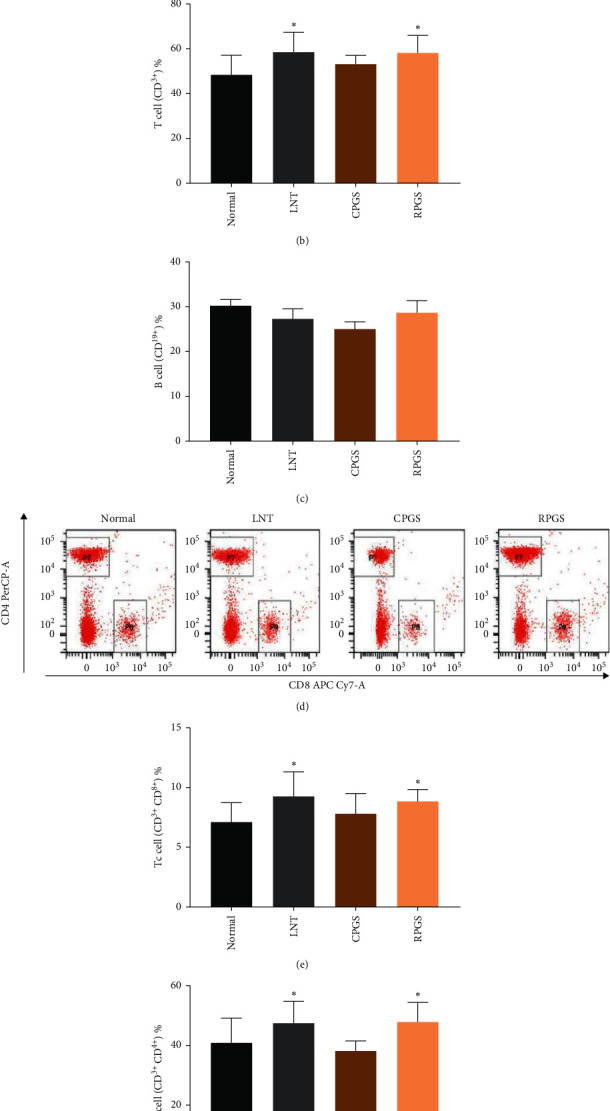
Effect of CPGS and RPGS on proportions of T-cells and B cells in peripheral blood. (a, b, c) Representative scatter diagram and quantitative analysis for T cells and B cells. (d, e, f) Representative scatter diagram and quantitative analysis for Th cells and Tc cells. Values were represented as means ± SD (*n* = 8–10).  ^*∗*^*p* < 0.05 and  ^*∗∗*^*p* < 0.01.

However, CPGS and RPGS had diverse effect on the splenocyte activity, including the splenocyte proliferation upon mitogen and the NK cell's tumor-killing ability. Results showed that upon the stimulation of mitogen (ConA or LPS), splenocyte proliferation was evidently suppressed by CPGS and RPGS treatment (Figures 1(d) and 1(e), *p* < 0.05), but the proliferation by LPS was not affected by LNT. CPGS showed an evident remarkable promotion on the NK cell's tumor-killing ability (Figure 2(f), *p* < 0.01), while RPGS did not affect it.

### 3.2. RPGS Modulated the Proportion of T and B Cells and Increased the Expression of CD28 in T cells in peripheral blood

**Figure 3:**
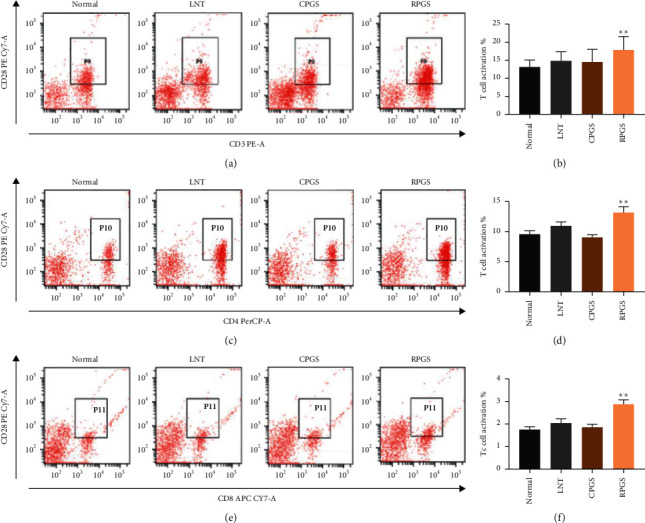
Effect of CPGS and RPGS on costimulatory factor CD28 of T-cell and T-cell subsets in peripheral blood. (a, b) Costimulatory factor CD28 in T-cells. (c, d) Costimulatory factor CD28 in Th cells. (e, f) Costimulatory factor CD28 in Tc cells. Values were represented as means ± SD (*n* = 8–10).  ^*∗*^*p* < 0.05 and  ^*∗∗*^*p* < 0.01.

As shown in Figure 2(a)‐2(c), percentages of T cell (CD^3+^) in peripheral blood of LNT and RPGS group were obviously higher than those of the normal group (*p* < 0.05), while the percentage of B cell (CD^19+^) was not affected by any of them. Data from Figure 2(d)‐2(f) show that proportion of helper T cells (Th cells, CD^3+^CD^4+^) and cytotoxic T cells (Tc cells, CD^3+^CD^8+^) of LNT and RPGS group were obviously higher than those of normal group (*p* < 0.05). Moreover, expressions of the costimulatory factor CD^28^ in T cell and T-cell subsets (Th cells and Tc cells) of the RPGS group were significantly higher than those of the normal group (Figure 3, *p* < 0.01). These results indicate that RPGS would be able to promote the activation of T cells.

### 3.3. CPGS and RPGS Affected the Proportion of Naive T Cell and Effector T Cell in Thymus

**Figure 4:**
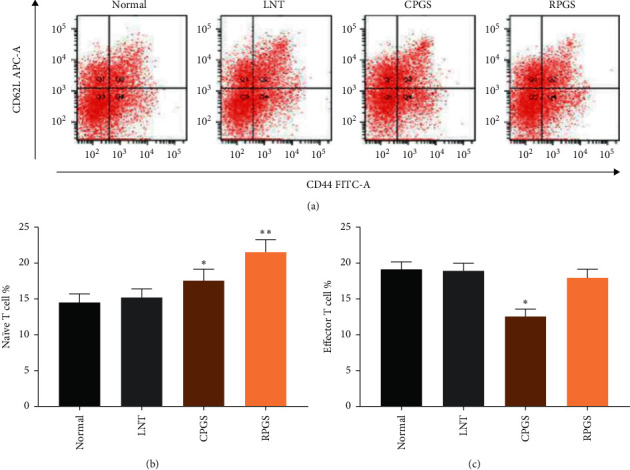
Effect of CPGS and RPGS on naïve T cells and effector T cells in the thymus, and T cells (a) Representative scatter diagram and quantitative analysis (b, c) for naïve T cells and effector T cells in the thymus. Values were represented as means ± SD (*n* = 8–10).  ^*∗*^*p* < 0.05 and  ^*∗∗*^*p* < 0.01.

Figure 4 showed that CPGS treatment evidently increased the percentage of naive T cell (CD^3+^CD^62L^CD^44+^^low^) (*p* < 0.05), while lowered that of effector T cell (CD^3+^CD^44+^CD^62Llow^) (*p* < 0.05). RPGS treatment displayed a more strong increase on naive T cell (*p* < 0.01), but did not affect effector T cell.

### 3.4. CPGS and RPGS Regulated the Composition and Function of Gut Microbiota

#### 3.4.1. *α*-Diversity and *β*-Diversity Analysis

**Figure 5:**
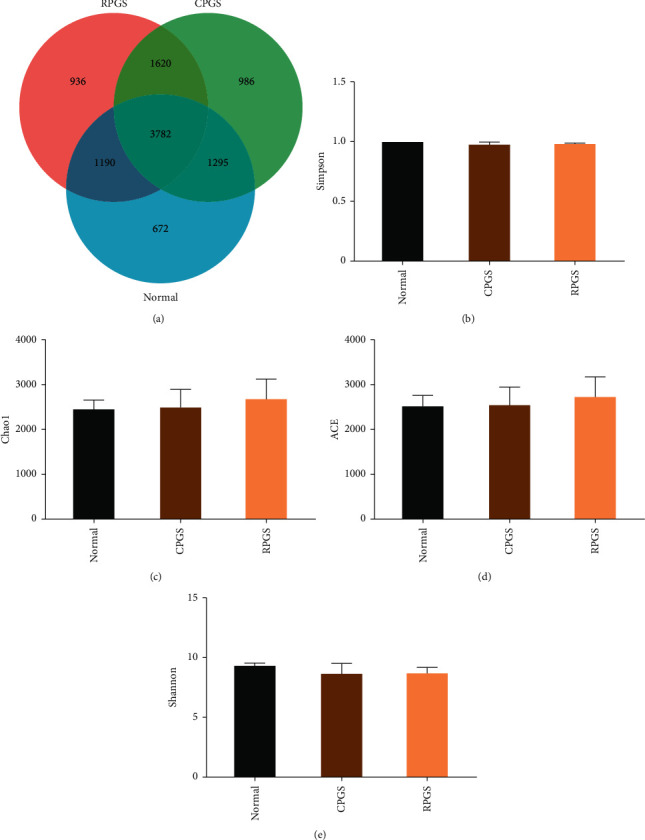
Venn diagram (a) and *α*-diversity index comparison (b–e). Values were represented as means ± SD (*n* = 8–10).  ^*∗*^*p* < 0.05 and  ^*∗∗*^*p* < 0.01.

Given that gut microbiota has been recognized as a pivotal assistant in chemotherapy and immunotherapy, we investigated its possible involvement in the activities of CPGS and RPGS in this part. A total of 10,483 sequences were obtained from all the fecal samples, with an average of 3,495 sequences per sample (Figure 5(a)). There existed 672 unique OTUs in the normal group, 936 in CPGS group, and 986 in RPGS group, respectively. 3782 common OTUs were shared by samples from all the three groups, and 1620 OTUs were only shared by CPGS and RPGS groups (Figure 5(a)).

*α*-Diversity analysis was applied to evaluate the richness and diversity of microbiota, including Simpson, Chao1, ACE, and Shannon. Chao1 and ACE were estimators for community richness [23, 24]. Shannon and Simpson's indices represent community diversity and uniformity. Data showed that (Figures 5(b)–5(e)) none of the indices was changed in the samples of CPGS and RPGS groups, demonstrating that CPGS and RPGS had no influence on community richness, diversity, or uniformity.

**Figure 6:**
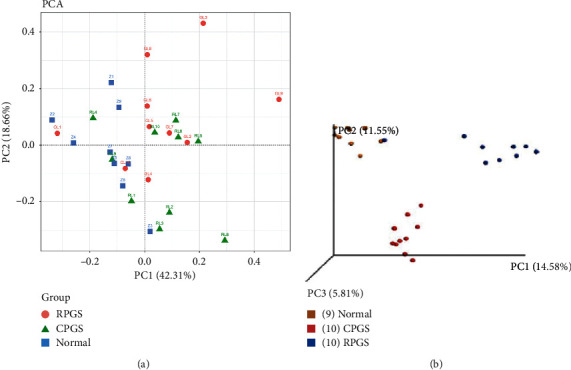
*β*-diversity analysis. (a) Principal component analysis (PCA). The percent variation explained by each principal coordinate was indicated on the axes. (b) Principle coordinates analysis (PCoA). The percent variation explained by each principal coordinate was indicated on the axes (*n* = 8–10).

*β*-Diversity analysis was used to compare the similarity of the overall community structure, including Principle Component Analysis (PCA), uniFrac distance-based principal coordinates analysis (PCoA), and analysis of similarities (ANOSIM). By PCA and PCoA, it was found that both microbiome structures of CPGS and RPGS were remarkably different from those of the normal group (Figure 6). The community structure difference was further confirmed by ANOSIM (Table 1), in which the unweighted R values of all group-wise comparisons were more than 0.7 (*p* < 0.01) and the weighted R values were 0.3∼0.4 (*p* < 0.01).

#### 3.4.2. Taxonomy Analysis and Correlation Analysis

**Figure 7:**
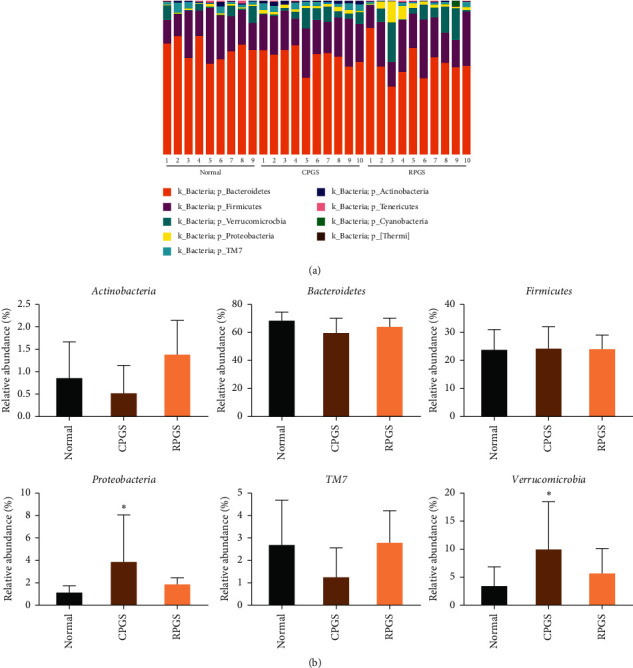
Taxonomy analysis of microbiota in the fecal at the phylum level. (a) Identified phyla in each sample. (b) Phylum relative abundance comparison (>0.1%). (c) Genera relative abundance comparison (0.1%). Values were represented as means ± SD (*n* = 8–10).  ^*∗*^*p* < 0.05 and  ^*∗∗*^*p* < 0.01.

**Figure 8:**
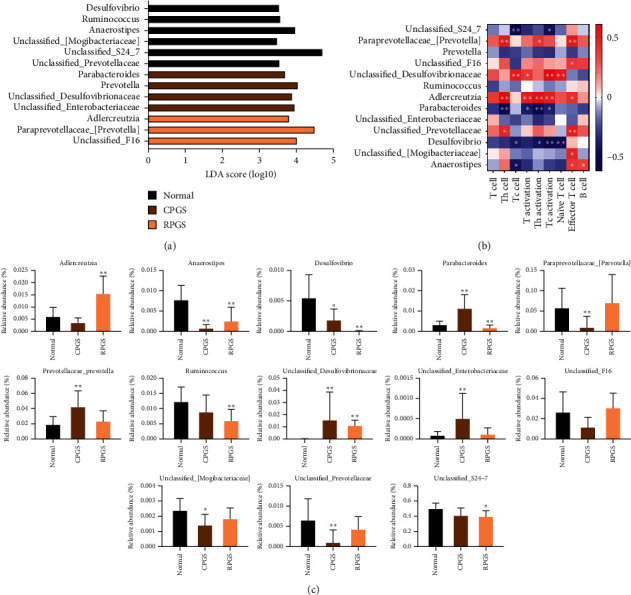
Taxonomy analysis of microbiota in the fecal at the genus level. (a) LDA scores results of the specifically enriched genera in each group. (b) Spearman analysis of microbiota-immune function presented by heatmap. (c) (b) Genus relative abundance comparison. Values were represented as means ± SD (*n* = 8–10).  ^*∗*^*p* < 0.05 and  ^*∗∗*^*p* < 0.01.

Taxonomy analysis revealed marked differences at both phylum and genus levels among normal, CPGS, and RPGS groups. Overall, a total of eight phyla were shared by samples from all groups (Figure 7(a), SHEET S1 Phylum). Of them, CPGS treatment significantly raised the relative abundances of *Verrucomicrobia* and *Proteobacteria*, but RPGS treatment increased that of *Actinobacteria* (Figure 7(b), *p* < 0.05). At the genus level, a total of 78 genera were identified from all samples (SHEET S2 Genus). LEfSe analysis was employed to figure out the enriched genus for each group (Figures 8(a) and 8(c)). It was found that among the genera with relative abundance median over 0.001%, *Anaerostipes*, *Desulfovibrio*, unclassified *Mogibacteriaceae*, unclassified *Prevotellaceae*, *Ruminococcus*, and unclassified *S24-7* were the highest in the normal group. By contrast, unclassified *Enterobacteriaceae*, unclassified *Desulfovibrionaceae, Parabacteroides*, and *Prevotellaceae*. *Prevotella* was enriched in the CPGS group, while *Adlercreutzia*, unclassified F16, and *Paraprevotellaceae* [*Prevotella*] were specific in the RPGS group. Moreover, it was found that there are certain correlations between immunoregulation and microbiota (Figure 8(b)). In particular, the enrichments of *Adlercreutzia* and unclassified *Desulfovibrionaceae* were positively correlated with T cell percentage and the activation of T cells, including Th cell and Tc cell. An increase of *Paraprevotellaceae* [*Prevotella*] was accompanied by the promotion of Th cell and its activation in peripheral blood, as well as effector T cells in thymus. Nevertheless, the relative abundance of *Parabacteroides* was negative related to T cell percentage and the activation of T cells.

#### 3.4.3. Prediction of Bacterial Metabolism

**Figure 9:**
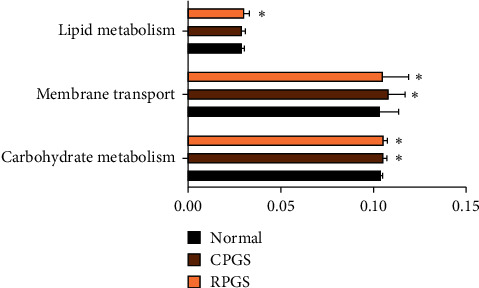
Metabolic pathway enrichment analysis. The predicted genes and their functions were aligned to the KEGG database, and the relative expressions for each pathway were compared. Values were represented as means ± SD (*n* = 8–10).  ^*∗*^*p* < 0.05 and  ^*∗∗*^*p* < 0.01.

By comparing the sequencing data with those collected in KEGG pathway database by PICRUSt (Figure 9), it was found that CPGS and RPGS were significantly upregulated the genes that are responsible for “carbohydrate metabolism,” “membrane transport,” and “lipid metabolism” (*p* < 0.05), indicating that together with the structural modulation, CPGS and RPGS could regulate the function of the gut microbiota to improve immunity.

## 4. Discussion

The immune system is a network system that is capable of monitoring, defense, and self-regulation. Generally, innate immunity and adaptive immunity comprise the entire immune system. Innate immunity is an evolutionary defense strategy [25] against pathogens and foreign objects. Adaptive immunity also referred as the acquired immune system. It can be divided into cell-mediated immunity and humoral immunity. The cell-mediated immunity responds to the presence of antigens and thus triggered cytotoxic T-lymphocytes and cytokines, while the humoral immunity relies on B-lymphocytes to produce antibodies for the purpose of countering attacks by antigens. Therefore, T cells and B cells are the major executors of adaptive immunity. In the present study, the immunomodulation by polysaccharide of sporoderm-broken spores of *G. lucidum* was explored from the perspective of the adaptive immune response.

The thymus is an important central immune organ where T cells develop, and the spleen is a key peripheral immune organ for lymphocyte storage and release, as well as for the development of immune memory. Naïve T cells (CD^3+^CD^62L^CD^44+low^), also known as unsensitized T cells, are enriched in thymus and are able to respond to novel pathogens that the immune system has not yet encountered. On the other hand, effector T cells (CD^3+^CD^44+^CD^62Llow^) are antigen-experienced naïve T cells that would provide an efficient cytotoxic activity. In the spleen, naïve T cells become activated and differentiate into memory/effector T cells once they are presented with the antigen-peptide-MHC-complexes by the antigen-loaded dendritic cells (DCs), and the immune response would be initiated upon the activation of appropriate costimulatory signals [26]. Data showed that both CPGS and RPGS increased the spleen coefficiency, but suppressed the proliferation upon mitogen stimulation; meanwhile, naïve T cell proportion in the thymus and the proportion of T cell in peripheral blood were increased. These results suggested that CPGS and RPGS posed diverse regulation on the adaptive immune, which would rely on the antigen recognition by naïve T cells.

T cells originate in the bone marrow and mature in the thymus. In the thymus, T cells multiply and differentiate into several subsets according to the difference of cell surface differentiation antigen (CD), including Th cells and Tc cells [27]. Th cells are available for the maturation of B cells into plasma cells and memory B cells, and the activation for Tc cells and macrophages. Tc cells destroy virus-infected cells and tumor cells and are also implicated in transplant rejection. T cell subsets interact and promote each other, at the same time restricting each other. The dynamic balance of the ratio of Th cells to Tc cells is the central hub of immune regulation [28]. CD28 (cluster of differentiation 28) is one of the proteins expressed on T cells that provide costimulatory signals required for T cell activation and survival. However, overexpression of costimulatory> molecules will make T cells overactivated, resulting in inflammation. Results showed that CPGS had no effect on the percentages of Th cells and Tc cells, neither on the expression of CD28. By contrast, RPGS significantly increased the subset proportion in peripheral blood and the expression of CD28, indicating that RPGS exhibited more prominent regulation on T cell-involving immunity.

Nowadays, the gut microbiota has been recognized as a regulator of both immune response and host metabolism [29]. For instance, high-fat diets change the gut microbiota composition by altering the *Firmicutes *: *Bacteroidetes* ratio and causing endotoxemia mainly by raising the levels of LPS [30]. And feces from the patients suffering from depression have fewer *Firmicutes* but more *Bacteroidetes*, *Proteobacteria*, and *Actinobacteria* [31]. In the present study, the *α*-diversity analysis indicated that treatment of CPGS and RPGS had no impact on community richness diversity or uniformity in comparison with that of normal group, while *β*-diversity analysis displayed evident structures difference among the overall communities of CPGS, RPGS, and normal group. By taxonomy analysis, it was found that both microbiotas of CPGS and RPGS groups had more unique microbes. Moreover, enrichments of *Adlercreutzia*, *Prevotella*, and the unclassified *Desulfovibrionaceae* were positively related to the immune regulation by CPGS and RPGS, including the percentage of T cell and the subsets, as well as their activation; while those of *Parabacteroides* displayed a negative correlation. *Adlercreutzia*, *Parabacteroides*, and *Prevotella* are phytoestrogen/polysaccharide-metabolizing bacteria, and they always have a positive relationship with immunity. For instance, *Adlercreutzia*, *Parabacteroides*, and *Prevotella* are higher in healthy control when compared with multiple sclerosis (MS) patients [32]. And a higher abundance of *Prevotella* is associated with favorable responses to antiprogrammed death 1 immunotherapy in Chinese patients with non-small-cell lung cancer (NSCLC) [33]. Indeed, the change of *Adlercreutzia* within the gut is revealed to be dependent on the type of polysaccharide. Cui et al. [34] found that marine plant-derived *Gelidium pacificumOkamura* polysaccharide decreased *Adlercreutzia*, while animal-derived *Cereus sinensis* polysaccharide increased it. *Parabacteroides* is found to be positively related with the attenuation by natural polysaccharide on myocardial injury induced by high-fat diet [35] and immune suppression induced by cyclophosphamide [36]. *Prevotella* spp. is a dominant bacterial genus within the human gut. *Prevotella* spp. exhibit variability in the utilization of diverse complex carbohydrates [37]. As revealed by several studies, *Prevotella* spp. would be promoted when the host takes in variable natural polysaccharides, which is a benefit for the amelioration of constipation symptoms [38], lipid metabolic disorders [39], and so on. The unclassified *Desulfovibrionaceae* was not detected in samples of the normal group, while it was evidently increased in samples of CPGS and RPGS groups. It has been revealed to be a positive outcome of the remission of colonic injury or inflammation [40, 41]. The promotion by CPGS and RPGS on these microbes suggested that polysaccharides of the *G. lucidum* spore endowed the mice with a unique gut microbiota structure, which is involved in its immunomodulation activity. In addition to the effect on community structure, it is noteworthy that the carbohydrate metabolism, membrane transport, and lipid metabolism pathways displayed distinct regulation upon the polysaccharide treatment. Taken together, it is probable that by oral supplement, polysaccharide of *G. lucidum* spores was able to increase the richness of beneficial microorganisms and regulates the microbiome function pathways, thereby improving the balance of adaptive immunity.

## 5. Conclusion

Collectively, the present study revealed that polysaccharides from *G. lucidum* spores would serve as a natural candidate for the improvement of the adaptive immune system, especially the refined polysaccharide. CPGS and RPGS exhibited diverse regulation on the adaptive immune response. The splenocyte proliferation activity upon mitogen was suppressed by CPGS and RPGS, while NK cell's tumor-killing ability was promoted by CPGS. Both CPGS and RPGS could increase the proportion of naïve T cells in the thymus, but only RPGS significantly uplifted the percentage of T cells, as well as the T cell subsets in peripheral blood, and promoted the activation, indicating that RPGS exhibit more prominent regulation on T cell-involving immunity. Moreover, the effects of CPGS and RPGS are closely related to the regulation on gut microbiota, which not only involves the changes in structure and community membership but also affects several metabolic pathways within the microbiome. And the enrichments of *Adlercreutzia*, *Prevotella*, and the unclassified *Desulfovibrionaceae* were positively related to the immune regulation by CPGS and RPGS. These findings suggested a promising effect of the polysaccharide from sporoderm-broken spores of *G. lucidum* in immune regulation to promote health control.

## Figures and Tables

**Table 1:** Result of the Analysis of similarities (ANOSIM, *n* = 8–10).

Group	Unweighted UniFrac		Weighted UniFrac	
	R statistic	*p* value	R statistic	*p* value
Normal vs CPGS	0.7877	0.001	0.3049	0.001
Normal vs RPGS	0.7852	0.001	0.3929	0.001
CPGS vs RPGS	0.7833	0.001	0.3374	0.002

## Data Availability

The data used to support the findings of this study are included within the article.

## Supplementary Materials

Supplementary Materials SHEET S1: relative abundance of phylum. SHEET S2: relative abundance of genus.
