# Supplementary material for: Polysaccharides of Sporoderm-Broken Spore of Ganoderma lucidum Modulate Adaptive Immune Function via Gut Microbiota Regulation
Source: Evid Based Complement Alternat Med. 2021 Mar 23;2021:8842062. doi: 10.1155/2021/8842062 (PMC8009716; doi:10.1155/2021/8842062)
Supplement: Supplementary Materials — SHEET S1: relative abundance of phylum. SHEET S2: relative abundance of genus. [file 8842062.f1.zip › 8842062.f1/SHEET S1 Phylum.docx]

Table S1- 1 Phylum relative abundance of Normal

| Taxon | Normal 1 | Normal 2 | Normal 3 | Normal 4 | Normal 5 | Normal 6 | Normal 7 | Normal 8 | Normal 9 |
| --- | --- | --- | --- | --- | --- | --- | --- | --- | --- |
| k__Bacteria;p__Bacteroidetes | 0.7198 | 0.767841 | 0.625738 | 0.77074 | 0.587631 | 0.619565 | 0.669251 | 0.71387 | 0.677212 |
| k__Bacteria;p__Firmicutes | 0.153285 | 0.144079 | 0.307195 | 0.164724 | 0.363688 | 0.284659 | 0.22861 | 0.225652 | 0.176924 |
| k__Bacteria;p__Verrucomicrobia | 0.097402 | 0.001857 | 0.004709 | 0.042986 | 0.008624 | 0.001989 | 0.068923 | 0.008387 | 0.110089 |
| k__Bacteria;p__Proteobacteria | 0.002244 | 0.007542 | 0.011291 | 0.003866 | 0.017929 | 0.010629 | 0.0116 | 0.007474 | 0.009836 |
| k__Bacteria;p__TM7 | 0.018572 | 0.065567 | 0.040683 | 0.005515 | 0.004823 | 0.0457 | 0.013751 | 0.020083 | 0.019671 |
| k__Bacteria;p__Actinobacteria | 0.002918 | 0.011031 | 0.007887 | 0.004037 | 0.004482 | 0.028761 | 0.003791 | 0.004393 | 0.005062 |
| k__Bacteria;p__Tenericutes | 0.005779 | 0.002082 | 0.002497 | 0.008017 | 0.012823 | 0.008583 | 0.004074 | 0.02014 | 0.00115 |
| k__Bacteria;p__Cyanobacteria | 0 | 0 | 0 | 0.000114 | 0 | 0.000114 | 0 | 0 | 0 |
| No blast hit;Other | 0 | 0 | 0 | 0 | 0 | 0 | 0 | 0 | 0 |
| k__Bacteria;p__[Thermi] | 0 | 0 | 0 | 0 | 0 | 0 | 0 | 0 | 5.75E-05 |

Table S1- 2 Phylum relative abundance of CPGS

| Taxon | CPGS 1 | CPGS 2 | CPGS 3 | CPGS 4 | CPGS 5 | CPGS 6 | CPGS 7 | CPGS 8 | CPGS 9 | CPGS 10 |
| --- | --- | --- | --- | --- | --- | --- | --- | --- | --- | --- |
| k__Bacteria;p__Bacteroidetes | 0.824132 | 0.569981 | 0.440564 | 0.53496 | 0.691257 | 0.494325 | 0.633104 | 0.594634 | 0.565203 | 0.576092 |
| k__Bacteria;p__Firmicutes | 0.143887 | 0.289713 | 0.159727 | 0.339014 | 0.22333 | 0.381378 | 0.258367 | 0.19906 | 0.178479 | 0.352219 |
| k__Bacteria;p__Verrucomicrobia | 0.005836 | 0.088385 | 0.257555 | 0.002887 | 0.037174 | 0.096932 | 0.061594 | 0.166176 | 0.205717 | 0.0092 |
| k__Bacteria;p__Proteobacteria | 0.00555 | 0.04528 | 0.136949 | 0.088037 | 0.030603 | 0.021353 | 0.020115 | 0.01664 | 0.010558 | 0.021148 |
| k__Bacteria;p__TM7 | 0.012815 | 0.003606 | 0.002071 | 0.021627 | 0.007319 | 0.003091 | 0.01591 | 0.014037 | 0 | 0.032367 |
| k__Bacteria;p__Actinobacteria | 0.003662 | 0.002977 | 0.000951 | 0.001982 | 0.002766 | 0.001686 | 0.002841 | 0.008377 | 0.005391 | 0.00359 |
| k__Bacteria;p__Tenericutes | 0.004119 | 5.72E-05 | 0.002183 | 0.011436 | 0.00755 | 0.001236 | 0.007955 | 0.000849 | 0 | 0.005385 |
| k__Bacteria;p__Cyanobacteria | 0 | 0 | 0 | 0 | 0 | 0 | 0 | 0 | 0.034651 | 0 |
| No blast hit;Other | 0 | 0 | 0 | 0 | 0 | 0 | 0.000114 | 0.00017 | 0 | 0 |
| k__Bacteria;p__[Thermi] | 0 | 0 | 0 | 5.66E-05 | 0 | 0 | 0 | 5.66E-05 | 0 | 0 |

Table S1- 3 Phylum relative abundance of RPGS

| Taxon | RPGS 1 | RPGS 2 | RPGS 3 | RPGS 4 | RPGS 5 | RPGS 6 | RPGS 7 | RPGS 8 | RPGS 9 | RPGS 10 |
| --- | --- | --- | --- | --- | --- | --- | --- | --- | --- | --- |
| k__Bacteria;p__Bacteroidetes | 0.67556 | 0.649735 | 0.676333 | 0.710354 | 0.499101 | 0.651384 | 0.658841 | 0.633822 | 0.5701 | 0.599682 |
| k__Bacteria;p__Firmicutes | 0.234834 | 0.248216 | 0.2585 | 0.171841 | 0.319996 | 0.220977 | 0.202302 | 0.255508 | 0.310689 | 0.247911 |
| k__Bacteria;p__Verrucomicrobia | 0.004582 | 0.0117 | 0.016056 | 0.045254 | 0.130887 | 0.077149 | 0.099118 | 0.041494 | 0.03371 | 0.075607 |
| k__Bacteria;p__Proteobacteria | 0.024475 | 0.016266 | 0.021889 | 0.015383 | 0.015005 | 0.014547 | 0.016949 | 0.028632 | 0.023899 | 0.008527 |
| k__Bacteria;p__TM7 | 0.043556 | 0.038354 | 0.002667 | 0.043184 | 0.028268 | 0.027113 | 0.012025 | 0.020412 | 0.043806 | 0.04417 |
| k__Bacteria;p__Actinobacteria | 0.014325 | 0.027396 | 0.024056 | 0.009453 | 0.005564 | 0.008434 | 0.010192 | 0.020132 | 0.015001 | 0.021431 |
| k__Bacteria;p__Tenericutes | 0.002494 | 0.008219 | 5.00E-04 | 0.004531 | 0.00118 | 0.000396 | 0.000573 | 0 | 0.002795 | 0.002672 |
| k__Bacteria;p__Cyanobacteria | 0.000174 | 0.000114 | 0 | 0 | 0 | 0 | 0 | 0 | 0 | 0 |
| No blast hit;Other | 0 | 0 | 0 | 0 | 0 | 0 | 0 | 0 | 0 | 0 |
| k__Bacteria;p__[Thermi] | 0 | 0 | 0 | 0 | 0 | 0 | 0 | 0 | 0 | 0 |
