# Supplementary material for: Polysaccharides of Sporoderm-Broken Spore of Ganoderma lucidum Modulate Adaptive Immune Function via Gut Microbiota Regulation
Source: Evid Based Complement Alternat Med. 2021 Mar 23;2021:8842062. doi: 10.1155/2021/8842062 (PMC8009716; doi:10.1155/2021/8842062)
Supplement: Supplementary Materials — SHEET S1: relative abundance of phylum. SHEET S2: relative abundance of genus. [file 8842062.f1.zip › 8842062.f1/SHEET S2 Genus.docx]

Table S2- 1 Genus relative abundance of Normal

| Taxon | Normal 1 | Normal 2 | Normal 3 | Normal 4 | Normal 5 | Normal 6 | Normal 7 | Normal 8 | Normal 9 |
| --- | --- | --- | --- | --- | --- | --- | --- | --- | --- |
| k__Bacteria;p__Bacteroidetes;c__Bacteroidia;o__Bacteroidales;f__S24-7;g__Unclassified_S24-7 | 0.536442 | 0.623086 | 0.355765 | 0.576676 | 0.467234 | 0.429887 | 0.484212 | 0.460261 | 0.498562 |
| k__Bacteria;p__Firmicutes;c__Clostridia;o__Clostridiales;f__Unclassified_Clostridiales;g__Unclassified_Clostridiales | 0.082814 | 0.076261 | 0.181514 | 0.052482 | 0.218667 | 0.145853 | 0.115211 | 0.089918 | 0.099965 |
| k__Bacteria;p__Bacteroidetes;c__Bacteroidia;o__Bacteroidales;f__Bacteroidaceae;g__Bacteroides | 0.093587 | 0.032812 | 0.042272 | 0.013533 | 0.062298 | 0.0241 | 0.011883 | 0.102699 | 0.052283 |
| k__Bacteria;p__Verrucomicrobia;c__Verrucomicrobiae;o__Verrucomicrobiales;f__Verrucomicrobiaceae;g__Akkermansia | 0.097402 | 0.001857 | 0.004709 | 0.042986 | 0.008624 | 0.001989 | 0.068923 | 0.008387 | 0.110089 |
| k__Bacteria;p__Bacteroidetes;c__Bacteroidia;o__Bacteroidales;f__[Paraprevotellaceae];g__[Prevotella] | 0.000617 | 0.025777 | 0.115695 | 0.132825 | 0.002213 | 0.049849 | 0.107345 | 0.029212 | 0.050673 |
| k__Bacteria;p__Firmicutes;c__Bacilli;o__Lactobacillales;f__Lactobacillaceae;g__Lactobacillus | 0.002918 | 0.005234 | 0.005731 | 0.034287 | 0.021277 | 0.052635 | 0.033952 | 0.002625 | 0.006039 |
| k__Bacteria;p__Bacteroidetes;c__Bacteroidia;o__Bacteroidales;f__Rikenellaceae;g__Unclassified_Rikenellaceae | 0.031645 | 0.043224 | 0.073479 | 0.017854 | 0.035291 | 0.08583 | 0.03067 | 0.074685 | 0.034913 |
| k__Bacteria;p__Firmicutes;c__Clostridia;o__Clostridiales;f__Lachnospiraceae;g__Unclassified_Lachnospiraceae | 0.017281 | 0.009455 | 0.037676 | 0.015921 | 0.030241 | 0.023475 | 0.022578 | 0.0554 | 0.022489 |
| k__Bacteria;p__Bacteroidetes;c__Bacteroidia;o__Bacteroidales;f__Prevotellaceae;g__Prevotella | 0.042249 | 0.024876 | 0.014866 | 0.006994 | 0.011404 | 0.008981 | 0.01194 | 0.021167 | 0.022374 |
| k__Bacteria;p__TM7;c__TM7-3;o__CW040;f__F16;g__Unclassified_F16 | 0.018572 | 0.065567 | 0.040683 | 0.005515 | 0.004823 | 0.0457 | 0.013751 | 0.020083 | 0.019671 |
| k__Bacteria;p__Firmicutes;c__Clostridia;o__Clostridiales;f__Ruminococcaceae;g__Unclassified_Ruminococcaceae | 0.013017 | 0.012945 | 0.023604 | 0.013192 | 0.025872 | 0.01745 | 0.014486 | 0.015633 | 0.013804 |
| k__Bacteria;p__Firmicutes;c__Clostridia;o__Clostridiales;f__Ruminococcaceae;g__Oscillospira | 0.010548 | 0.018404 | 0.019859 | 0.0087 | 0.019404 | 0.012334 | 0.011827 | 0.016375 | 0.010123 |
| k__Bacteria;p__Firmicutes;c__Erysipelotrichi;o__Erysipelotrichales;f__Erysipelotrichaceae;g__Unclassified_Erysipelotrichaceae | 0.005386 | 0.003039 | 0.016114 | 0.008756 | 0.00556 | 0.009322 | 0.003112 | 0.017573 | 0.003969 |
| k__Bacteria;p__Bacteroidetes;c__Bacteroidia;o__Bacteroidales;f__Unclassified_Bacteroidales;g__Unclassified_Bacteroidales | 0.00735 | 0.009568 | 0.011235 | 0.004947 | 0.006638 | 0.013301 | 0.007356 | 0.011982 | 0.007305 |
| k__Bacteria;p__Proteobacteria;c__Deltaproteobacteria;o__Desulfovibrionales;f__Desulfovibrionaceae;g__Unclassified_Desulfovibrionaceae | 0 | 0 | 0 | 0.000171 | 0 | 0 | 0 | 0 | 0 |
| k__Bacteria;p__Firmicutes;c__Clostridia;o__Clostridiales;f__Ruminococcaceae;g__Ruminococcus | 0.011783 | 0.003883 | 0.010497 | 0.016376 | 0.02105 | 0.01188 | 0.015278 | 0.009528 | 0.008513 |
| k__Bacteria;p__Actinobacteria;c__Coriobacteriia;o__Coriobacteriales;f__Coriobacteriaceae;g__Adlercreutzia | 0.002918 | 0.008273 | 0.007547 | 0.0029 | 0.003972 | 0.015233 | 0.003395 | 0.004279 | 0.004831 |
| k__Bacteria;p__Firmicutes;c__Clostridia;o__Clostridiales;f__Lachnospiraceae;g__[Ruminococcus] | 0.00202 | 0.003715 | 0.001419 | 0.006198 | 0.008681 | 0.002785 | 0.003339 | 0.002225 | 0.002473 |
| k__Bacteria;p__Bacteroidetes;c__Bacteroidia;o__Bacteroidales;f__Porphyromonadaceae;g__Parabacteroides | 0.004208 | 0.002758 | 0.001419 | 0.001933 | 0.001418 | 0.001592 | 0.002377 | 0.007246 | 0.004429 |
| k__Bacteria;p__Proteobacteria;c__Gammaproteobacteria;o__Enterobacteriales;f__Enterobacteriaceae;g__Unclassified_Enterobacteriaceae | 0 | 0 | 0.00017 | 0.000114 | 0 | 0.000227 | 0 | 0 | 0.00023 |
| k__Bacteria;p__Tenericutes;c__Mollicutes;o__RF39;f__Unclassified_RF39;g__Unclassified_RF39 | 0.005779 | 0.002082 | 0.002383 | 0.007562 | 0.012823 | 0.008583 | 0.004074 | 0.02014 | 0.00115 |
| k__Bacteria;p__Bacteroidetes;c__Bacteroidia;o__Bacteroidales;f__Prevotellaceae;g__Unclassified_Prevotellaceae | 0.000112 | 0.00529 | 0.010611 | 0.015295 | 0.00034 | 0.004263 | 0.012675 | 0.00348 | 0.006615 |
| k__Bacteria;p__Proteobacteria;c__Deltaproteobacteria;o__Desulfovibrionales;f__Desulfovibrionaceae;g__Desulfovibrio | 0.000617 | 0.004052 | 0.004823 | 0.001308 | 0.012709 | 0.005514 | 0.009959 | 0.003766 | 0.006442 |
| k__Bacteria;p__Firmicutes;c__Clostridia;o__Clostridiales;f__Lachnospiraceae;g__Coprococcus | 0.000673 | 0.001745 | 0.004483 | 0.001194 | 0.00278 | 0.002274 | 0.003735 | 0.001712 | 0.002416 |
| k__Bacteria;p__Firmicutes;c__Clostridia;o__Clostridiales;f__Dehalobacteriaceae;g__Dehalobacterium | 0.000337 | 0.001801 | 0.001645 | 0.000114 | 0.000738 | 0.001705 | 0.000736 | 0.002796 | 0.001323 |
| k__Bacteria;p__Firmicutes;c__Clostridia;o__Clostridiales;f__[Mogibacteriaceae];g__Unclassified_[Mogibacteriaceae] | 0.001795 | 0.002476 | 0.001248 | 0.003014 | 0.003688 | 0.001364 | 0.002094 | 0.002739 | 0.002761 |
| k__Bacteria;p__Proteobacteria;c__Gammaproteobacteria;o__Pseudomonadales;f__Moraxellaceae;g__Unclassified_Moraxellaceae | 0.000281 | 0.001126 | 0.001419 | 0.000796 | 0.00017 | 0.001648 | 0.000396 | 0.000685 | 0.00115 |
| k__Bacteria;p__Proteobacteria;c__Epsilonproteobacteria;o__Campylobacterales;f__Helicobacteraceae;g__Unclassified_Helicobacteraceae | 0.000617 | 0.000507 | 0 | 0.000398 | 0.003007 | 0.000512 | 0.000453 | 0.000856 | 0.000518 |
| k__Bacteria;p__Proteobacteria;c__Gammaproteobacteria;o__Pseudomonadales;f__Pseudomonadaceae;g__Pseudomonas | 0.000337 | 0.001013 | 0.001759 | 0.000853 | 0.000284 | 0.000966 | 0.000283 | 0.001312 | 0.000978 |
| k__Bacteria;p__Firmicutes;c__Clostridia;o__Clostridiales;f__Lachnospiraceae;g__Dorea | 0 | 0.001238 | 0.000454 | 0.000398 | 0.000454 | 0.000227 | 0.000566 | 0.001655 | 0.000518 |
| k__Bacteria;p__Cyanobacteria;c__4C0d-2;o__YS2;f__Unclassified_YS2;g__Unclassified_YS2 | 0 | 0 | 0 | 0 | 0 | 0 | 0 | 0 | 0 |
| k__Bacteria;p__Proteobacteria;c__Epsilonproteobacteria;o__Campylobacterales;f__Helicobacteraceae;g__Helicobacter | 0 | 0 | 0 | 0 | 0.000284 | 0 | 0 | 0 | 0 |
| k__Bacteria;p__Actinobacteria;c__Actinobacteria;o__Bifidobacteriales;f__Bifidobacteriaceae;g__Bifidobacterium | 0 | 0.002758 | 0.000113 | 0.001137 | 0.000284 | 0.01296 | 0.000396 | 0.000114 | 0.00023 |
| k__Bacteria;p__Firmicutes;c__Clostridia;o__Clostridiales;f__Clostridiaceae;g__Clostridium | 0.000786 | 0.001013 | 0.000284 | 0.000284 | 0.001418 | 0.000853 | 0.000113 | 0.000456 | 0.000173 |
| k__Bacteria;p__Proteobacteria;c__Deltaproteobacteria;o__Desulfovibrionales;f__Desulfovibrionaceae;g__Bilophila | 0.000112 | 0.000225 | 0.00278 | 0 | 0.001191 | 0.001364 | 0.00017 | 0 | 0.000518 |
| k__Bacteria;p__Firmicutes;c__Bacilli;o__Lactobacillales;f__Streptococcaceae;g__Streptococcus | 0.000281 | 0.00045 | 0 | 0 | 0.000113 | 0.000171 | 0.000113 | 0.000171 | 0 |
| k__Bacteria;p__Bacteroidetes;c__Bacteroidia;o__Bacteroidales;f__Rikenellaceae;g__AF12 | 0.000561 | 0.00045 | 0.000397 | 0.000227 | 0.000794 | 0 | 0.00017 | 0.003138 | 5.75E-05 |
| k__Bacteria;p__Firmicutes;c__Clostridia;o__Clostridiales;f__Lachnospiraceae;g__Anaerostipes | 0.000898 | 0.000675 | 0.001248 | 0.000114 | 0.001191 | 0.000625 | 0.000679 | 0.001027 | 0.000403 |
| k__Bacteria;p__Firmicutes;c__Bacilli;o__Lactobacillales;f__Leuconostocaceae;g__Unclassified_Leuconostocaceae | 0 | 0 | 0 | 0 | 0 | 0 | 0 | 0 | 0 |
| k__Bacteria;p__Proteobacteria;c__Gammaproteobacteria;o__Vibrionales;f__Vibrionaceae;g__Vibrio | 0 | 0 | 0 | 0 | 0 | 0 | 0 | 0 | 0 |
| k__Bacteria;p__Firmicutes;c__Clostridia;o__Clostridiales;f__Clostridiaceae;g__Unclassified_Clostridiaceae | 0.000673 | 0 | 0.00017 | 0.000398 | 0.000624 | 0.000284 | 0.00034 | 0.000456 | 0.000403 |
| k__Bacteria;p__Firmicutes;c__Clostridia;o__Clostridiales;f__Lachnospiraceae;g__Roseburia | 0.000673 | 0.000169 | 0.000284 | 0 | 0.000284 | 0.000512 | 5.66E-05 | 0.004222 | 0.000115 |
| k__Bacteria;p__Firmicutes;c__Clostridia;o__Clostridiales;f__Lachnospiraceae;g__Blautia | 0.000786 | 0 | 0.000227 | 0.000171 | 0.000567 | 0.000114 | 0 | 0 | 0 |
| k__Bacteria;p__Firmicutes;c__Clostridia;o__Clostridiales;f__Ruminococcaceae;g__Faecalibacterium | 0.000112 | 0.0009 | 0.00017 | 0.00199 | 0 | 0 | 0 | 0.000228 | 0.00023 |
| k__Bacteria;p__Bacteroidetes;c__Bacteroidia;o__Bacteroidales;f__[Odoribacteraceae];g__Odoribacter | 0.002918 | 0 | 0 | 0.000455 | 0 | 0.001592 | 0.000622 | 0 | 0 |
| k__Bacteria;p__Firmicutes;c__Erysipelotrichi;o__Erysipelotrichales;f__Erysipelotrichaceae;g__Coprobacillus | 0 | 0 | 0.000227 | 0.000114 | 0 | 0.000171 | 0.00017 | 0.000114 | 0.000288 |
| k__Bacteria;p__Firmicutes;c__Clostridia;o__Clostridiales;f__Christensenellaceae;g__Unclassified_Christensenellaceae | 0.000224 | 0.000675 | 0.00017 | 0.000114 | 0.000567 | 0.000227 | 0.000113 | 0.000228 | 0.00046 |
| k__Bacteria;p__Proteobacteria;c__Alphaproteobacteria;o__Unclassified_Alphaproteobacteria;f__Unclassified_Alphaproteobacteria;g__Unclassified_Alphaproteobacteria | 0.000281 | 0 | 0 | 0 | 0.000113 | 0 | 0.00034 | 0.000114 | 0 |
| k__Bacteria;p__Firmicutes;c__Clostridia;o__Clostridiales;f__Clostridiaceae;g__Candidatus_Arthromitus | 0.000281 | 0 | 0.00017 | 0.000512 | 0.000113 | 0 | 0 | 0.000228 | 0.000115 |
| k__Bacteria;p__Firmicutes;c__Clostridia;o__Clostridiales;f__Ruminococcaceae;g__Anaerotruncus | 0 | 0 | 0 | 0.000114 | 0.00017 | 0 | 0.000113 | 5.71E-05 | 0 |
| k__Bacteria;p__Proteobacteria;c__Gammaproteobacteria;o__Pseudomonadales;f__Pseudomonadaceae;g__Unclassified_Pseudomonadaceae | 0 | 0 | 0 | 0 | 0 | 0 | 0 | 0 | 0 |
| k__Bacteria;p__Firmicutes;c__Clostridia;o__Clostridiales;f__Lachnospiraceae;g__Pseudobutyrivibrio | 0 | 0 | 0 | 0 | 0 | 0.000227 | 0 | 0 | 0.000115 |
| k__Bacteria;p__Proteobacteria;c__Alphaproteobacteria;o__Rhizobiales;f__Brucellaceae;g__Ochrobactrum | 0 | 0.000281 | 0.000227 | 0 | 0.00017 | 0.000171 | 0 | 0.000114 | 0 |
| k__Bacteria;p__Firmicutes;c__Erysipelotrichi;o__Erysipelotrichales;f__Erysipelotrichaceae;g__[Eubacterium] | 0 | 0 | 0 | 0 | 0.000227 | 0 | 0 | 0 | 0 |
| k__Bacteria;p__Firmicutes;c__Bacilli;o__Lactobacillales;f__Unclassified_Lactobacillales;g__Unclassified_Lactobacillales | 0 | 0 | 0 | 0 | 0 | 0 | 0 | 0 | 0 |
| k__Bacteria;p__Tenericutes;c__Mollicutes;o__Anaeroplasmatales;f__Anaeroplasmataceae;g__Anaeroplasma | 0 | 0 | 0.000113 | 0.000455 | 0 | 0 | 0 | 0 | 0 |
| k__Bacteria;p__Firmicutes;c__Bacilli;o__Bacillales;f__Planococcaceae;g__Staphylococcus | 0 | 0 | 0 | 0 | 0 | 0 | 0 | 0 | 0 |
| k__Bacteria;p__Actinobacteria;c__Coriobacteriia;o__Coriobacteriales;f__Coriobacteriaceae;g__Unclassified_Coriobacteriaceae | 0 | 0 | 0.000113 | 0 | 0.000113 | 0.000114 | 0 | 0 | 0 |
| k__Bacteria;p__Firmicutes;c__Clostridia;o__Clostridiales;f__Eubacteriaceae;g__Anaerofustis | 0 | 0 | 0 | 0.000171 | 0 | 0 | 0 | 5.71E-05 | 0 |
| k__Bacteria;p__Proteobacteria;c__Alphaproteobacteria;o__RF32;f__Unclassified_RF32;g__Unclassified_RF32 | 0 | 0 | 0 | 0 | 0 | 0 | 0 | 0.000571 | 0 |
| k__Bacteria;p__Proteobacteria;c__Gammaproteobacteria;o__Pseudomonadales;f__Moraxellaceae;g__Acinetobacter | 0 | 0 | 0 | 0 | 0 | 0 | 0 | 0 | 0 |
| k__Bacteria;p__Proteobacteria;c__Gammaproteobacteria;o__Enterobacteriales;f__Enterobacteriaceae;g__Proteus | 0 | 0 | 0 | 0.000114 | 0 | 0 | 0 | 0 | 0 |
| k__Bacteria;p__Firmicutes;c__Bacilli;o__Lactobacillales;f__Enterococcaceae;g__Enterococcus | 0 | 0 | 0 | 0 | 0 | 0 | 0 | 0 | 0 |
| k__Bacteria;p__Bacteroidetes;c__Bacteroidia;o__Bacteroidales;f__[Barnesiellaceae];g__Unclassified_[Barnesiellaceae] | 0.000112 | 0 | 0 | 0 | 0 | 0 | 0 | 0 | 0 |
| k__Bacteria;p__Actinobacteria;c__Actinobacteria;o__Actinomycetales;f__Micrococcaceae;g__Rothia | 0 | 0 | 0 | 0 | 0.000113 | 0.000455 | 0 | 0 | 0 |
| k__Bacteria;p__Firmicutes;c__Clostridia;o__Clostridiales;f__Peptococcaceae;g__Unclassified_Peptococcaceae | 0 | 0 | 0 | 0 | 0 | 0.000171 | 0 | 0.000171 | 0.00023 |
| k__Bacteria;p__Firmicutes;c__Bacilli;o__Bacillales;f__Staphylococcaceae;g__Staphylococcus | 0 | 0 | 0 | 0.000114 | 0 | 0 | 0 | 0 | 0 |
| k__Bacteria;p__Proteobacteria;c__Epsilonproteobacteria;o__Campylobacterales;f__Helicobacteraceae;g__Flexispira | 0 | 0 | 0 | 0 | 0 | 0 | 0 | 0 | 0 |
| k__Bacteria;p__Proteobacteria;c__Betaproteobacteria;o__Burkholderiales;f__Alcaligenaceae;g__Sutterella | 0 | 0.000225 | 0 | 0.000114 | 0 | 0 | 0 | 0 | 0 |
| k__Bacteria;p__Cyanobacteria;c__Chloroplast;o__Streptophyta;f__Unclassified_Streptophyta;g__Unclassified_Streptophyta | 0 | 0 | 0 | 0.000114 | 0 | 0.000114 | 0 | 0 | 0 |
| k__Bacteria;p__Actinobacteria;c__Actinobacteria;o__Actinomycetales;f__Pseudonocardiaceae;g__Amycolatopsis | 0 | 0 | 0.000113 | 0 | 0 | 0 | 0 | 0 | 0 |
| k__Bacteria;p__Proteobacteria;c__Gammaproteobacteria;o__Xanthomonadales;f__Xanthomonadaceae;g__Unclassified_Xanthomonadaceae | 0 | 0.000113 | 0 | 0 | 0 | 0 | 0 | 5.71E-05 | 0 |
| k__Bacteria;p__Bacteroidetes;c__[Saprospirae];o__[Saprospirales];f__Chitinophagaceae;g__Sediminibacterium | 0 | 0 | 0 | 0 | 0 | 0.000171 | 0 | 0 | 0 |
| k__Bacteria;p__Firmicutes;c__Erysipelotrichi;o__Erysipelotrichales;f__Erysipelotrichaceae;g__Clostridium | 0 | 0 | 0 | 0 | 0 | 0 | 0 | 5.71E-05 | 0 |
| k__Bacteria;p__Proteobacteria;c__Betaproteobacteria;o__Neisseriales;f__Neisseriaceae;g__Neisseria | 0 | 0 | 0 | 0 | 0 | 0.000227 | 0 | 0 | 0 |
| k__Bacteria;p__Proteobacteria;c__Betaproteobacteria;o__Burkholderiales;f__Oxalobacteraceae;g__Cupriavidus | 0 | 0 | 0.000113 | 0 | 0 | 0 | 0 | 0 | 0 |
| No blast hit;Other;Other;Other;Other;Other | 0 | 0 | 0 | 0 | 0 | 0 | 0 | 0 | 0 |
| k__Bacteria;p__[Thermi];c__Deinococci;o__Thermales;f__Thermaceae;g__Thermus | 0 | 0 | 0 | 0 | 0 | 0 | 0 | 0 | 5.75E-05 |

Table S2- 2 Genus relative abundance of CPGS

| Taxon | CPGS 1 | CPGS 2 | CPGS 3 | CPGS 4 | CPGS 5 | CPGS 6 | CPGS 7 | CPGS 8 | CPGS 9 | CPGS 10 |
| --- | --- | --- | --- | --- | --- | --- | --- | --- | --- | --- |
| k__Bacteria;p__Bacteroidetes;c__Bacteroidia;o__Bacteroidales;f__S24-7;g__Unclassified_S24-7 | 0.615596 | 0.346156 | 0.339154 | 0.38725 | 0.443836 | 0.421612 | 0.40042 | 0.445212 | 0.215152 | 0.448084 |
| k__Bacteria;p__Firmicutes;c__Clostridia;o__Clostridiales;f__Unclassified_Clostridiales;g__Unclassified_Clostridiales | 0.06196 | 0.102982 | 0.055294 | 0.153202 | 0.064722 | 0.107215 | 0.083812 | 0.061467 | 0.020555 | 0.171313 |
| k__Bacteria;p__Bacteroidetes;c__Bacteroidia;o__Bacteroidales;f__Bacteroidaceae;g__Bacteroides | 0.033869 | 0.100521 | 0.011697 | 0.051633 | 0.140107 | 0.007136 | 0.143645 | 0.017263 | 0.224026 | 0.055253 |
| k__Bacteria;p__Verrucomicrobia;c__Verrucomicrobiae;o__Verrucomicrobiales;f__Verrucomicrobiaceae;g__Akkermansia | 0.005836 | 0.088385 | 0.257555 | 0.002887 | 0.037174 | 0.096932 | 0.061594 | 0.166176 | 0.205717 | 0.0092 |
| k__Bacteria;p__Bacteroidetes;c__Bacteroidia;o__Bacteroidales;f__[Paraprevotellaceae];g__[Prevotella] | 0.090623 | 0.000114 | 0 | 5.66E-05 | 0 | 0 | 0 | 0 | 0 | 0.000393 |
| k__Bacteria;p__Firmicutes;c__Bacilli;o__Lactobacillales;f__Lactobacillaceae;g__Lactobacillus | 0.048401 | 0.025016 | 0.066711 | 0.021684 | 0.091234 | 0.186165 | 0.069379 | 0.070297 | 0.025048 | 0.002019 |
| k__Bacteria;p__Bacteroidetes;c__Bacteroidia;o__Bacteroidales;f__Rikenellaceae;g__Unclassified_Rikenellaceae | 0.018651 | 0.05942 | 0.024681 | 0.03329 | 0.035502 | 0.01933 | 0.036423 | 0.018848 | 0.027743 | 0.037583 |
| k__Bacteria;p__Firmicutes;c__Clostridia;o__Clostridiales;f__Lachnospiraceae;g__Unclassified_Lachnospiraceae | 0.00944 | 0.087183 | 0.004309 | 0.083395 | 0.019423 | 0.046078 | 0.040855 | 0.015282 | 0.047344 | 0.093117 |
| k__Bacteria;p__Bacteroidetes;c__Bacteroidia;o__Bacteroidales;f__Prevotellaceae;g__Prevotella | 0.049316 | 0.034518 | 0.03912 | 0.022929 | 0.056539 | 0.033603 | 0.037957 | 0.094634 | 0.02078 | 0.025804 |
| k__Bacteria;p__TM7;c__TM7-3;o__CW040;f__F16;g__Unclassified_F16 | 0.012815 | 0.003606 | 0.002071 | 0.021627 | 0.007319 | 0.003091 | 0.01591 | 0.014037 | 0 | 0.032367 |
| k__Bacteria;p__Firmicutes;c__Clostridia;o__Clostridiales;f__Ruminococcaceae;g__Unclassified_Ruminococcaceae | 0.008582 | 0.018662 | 0.005205 | 0.022759 | 0.007953 | 0.009497 | 0.014944 | 0.009905 | 0.004886 | 0.025243 |
| k__Bacteria;p__Firmicutes;c__Clostridia;o__Clostridiales;f__Ruminococcaceae;g__Oscillospira | 0.005035 | 0.0162 | 0.006604 | 0.020325 | 0.010086 | 0.010845 | 0.01108 | 0.00549 | 0.015163 | 0.009312 |
| k__Bacteria;p__Firmicutes;c__Erysipelotrichi;o__Erysipelotrichales;f__Erysipelotrichaceae;g__Unclassified_Erysipelotrichaceae | 0.002117 | 0.007957 | 0.006996 | 0.003906 | 0.012103 | 0.001236 | 0.015683 | 0.004981 | 0.039088 | 0.006675 |
| k__Bacteria;p__Bacteroidetes;c__Bacteroidia;o__Bacteroidales;f__Unclassified_Bacteroidales;g__Unclassified_Bacteroidales | 0.004005 | 0.007385 | 0.00666 | 0.021061 | 0.008011 | 0.003372 | 0.005966 | 0.005207 | 0.064697 | 0.006787 |
| k__Bacteria;p__Proteobacteria;c__Deltaproteobacteria;o__Desulfovibrionales;f__Desulfovibrionaceae;g__Unclassified_Desulfovibrionaceae | 0.000286 | 0.032515 | 0.001847 | 0.075921 | 0.0034 | 0.01298 | 0.005114 | 0.00566 | 0.000899 | 0.012509 |
| k__Bacteria;p__Firmicutes;c__Clostridia;o__Clostridiales;f__Ruminococcaceae;g__Ruminococcus | 0.002746 | 0.006984 | 0.003526 | 0.020325 | 0.006858 | 0.004776 | 0.008523 | 0.010358 | 0.006515 | 0.01666 |
| k__Bacteria;p__Actinobacteria;c__Coriobacteriia;o__Coriobacteriales;f__Coriobacteriaceae;g__Adlercreutzia | 0.003662 | 0.002977 | 0.000839 | 0.001982 | 0.002594 | 0.001686 | 0.002841 | 0.008377 | 0.005391 | 0.003478 |
| k__Bacteria;p__Firmicutes;c__Clostridia;o__Clostridiales;f__Lachnospiraceae;g__[Ruminococcus] | 0.00143 | 0.010018 | 0.000504 | 0.005492 | 0.002075 | 0.007755 | 0.004489 | 0.002943 | 0.003201 | 0.016885 |
| k__Bacteria;p__Bacteroidetes;c__Bacteroidia;o__Bacteroidales;f__Porphyromonadaceae;g__Parabacteroides | 0.001888 | 0.021753 | 0.019252 | 0.01874 | 0.007262 | 0.009272 | 0.00858 | 0.013188 | 0.008873 | 0.002188 |
| k__Bacteria;p__Proteobacteria;c__Gammaproteobacteria;o__Enterobacteriales;f__Enterobacteriaceae;g__Unclassified_Enterobacteriaceae | 0.000286 | 0.001889 | 0.131072 | 0.000793 | 0 | 0.000281 | 0 | 0.00532 | 5.62E-05 | 0.000617 |
| k__Bacteria;p__Tenericutes;c__Mollicutes;o__RF39;f__Unclassified_RF39;g__Unclassified_RF39 | 0.004119 | 5.72E-05 | 0.002183 | 0.01138 | 0.00755 | 0.001236 | 0.007841 | 0.000736 | 0 | 0.005385 |
| k__Bacteria;p__Bacteroidetes;c__Bacteroidia;o__Bacteroidales;f__Prevotellaceae;g__Unclassified_Prevotellaceae | 0.010012 | 0 | 0 | 0 | 0 | 0 | 0 | 0 | 0 | 0 |
| k__Bacteria;p__Proteobacteria;c__Deltaproteobacteria;o__Desulfovibrionales;f__Desulfovibrionaceae;g__Desulfovibrio | 0.000629 | 0.003034 | 0 | 0.003963 | 0.000461 | 0.001292 | 0.002386 | 0.00017 | 0.000337 | 0.005553 |
| k__Bacteria;p__Firmicutes;c__Clostridia;o__Clostridiales;f__Lachnospiraceae;g__Coprococcus | 0.000458 | 0.002118 | 0.00028 | 0.002661 | 0.000403 | 0.00472 | 0.000909 | 5.66E-05 | 0.000562 | 0.002076 |
| k__Bacteria;p__Firmicutes;c__Clostridia;o__Clostridiales;f__Dehalobacteriaceae;g__Dehalobacterium | 0.000515 | 0.004866 | 5.60E-05 | 0.002774 | 0.002536 | 0.000562 | 0.002386 | 0 | 0.004324 | 0.002805 |
| k__Bacteria;p__Firmicutes;c__Clostridia;o__Clostridiales;f__[Mogibacteriaceae];g__Unclassified_[Mogibacteriaceae] | 0.001316 | 0.00166 | 0.001231 | 0.001132 | 0.002017 | 0.000393 | 0.003068 | 0.000849 | 0.001236 | 0.000898 |
| k__Bacteria;p__Proteobacteria;c__Gammaproteobacteria;o__Pseudomonadales;f__Moraxellaceae;g__Unclassified_Moraxellaceae | 0.001316 | 0.002519 | 0.001119 | 0.003001 | 0.002939 | 0.001573 | 0.000739 | 0.003056 | 0.00073 | 0.000449 |
| k__Bacteria;p__Proteobacteria;c__Epsilonproteobacteria;o__Campylobacterales;f__Helicobacteraceae;g__Unclassified_Helicobacteraceae | 0.001888 | 0.002576 | 0.000448 | 0.001415 | 0.003516 | 0.003877 | 0.0025 | 0.000283 | 0.002359 | 0.000337 |
| k__Bacteria;p__Proteobacteria;c__Gammaproteobacteria;o__Pseudomonadales;f__Pseudomonadaceae;g__Pseudomonas | 0.000801 | 0.001145 | 0.001007 | 0.002151 | 0.001153 | 0.001011 | 0.000455 | 0.001698 | 0.000393 | 0.000841 |
| k__Bacteria;p__Firmicutes;c__Clostridia;o__Clostridiales;f__Lachnospiraceae;g__Dorea | 0 | 0.000572 | 0.006324 | 0 | 0.000403 | 0.001124 | 0.000568 | 0.010867 | 0.002696 | 0 |
| k__Bacteria;p__Cyanobacteria;c__4C0d-2;o__YS2;f__Unclassified_YS2;g__Unclassified_YS2 | 0 | 0 | 0 | 0 | 0 | 0 | 0 | 0 | 0.034651 | 0 |
| k__Bacteria;p__Proteobacteria;c__Epsilonproteobacteria;o__Campylobacterales;f__Helicobacteraceae;g__Helicobacter | 0.000229 | 5.72E-05 | 0 | 0 | 0.018327 | 0 | 0.00841 | 0 | 0.003931 | 0 |
| k__Bacteria;p__Actinobacteria;c__Actinobacteria;o__Bifidobacteriales;f__Bifidobacteriaceae;g__Bifidobacterium | 0 | 0 | 0.000112 | 0 | 0 | 0 | 0 | 0 | 0 | 0 |
| k__Bacteria;p__Firmicutes;c__Clostridia;o__Clostridiales;f__Clostridiaceae;g__Clostridium | 0.000172 | 0.000572 | 0.000224 | 5.66E-05 | 0.000115 | 0.000337 | 0.000114 | 0.000453 | 0 | 0.001739 |
| k__Bacteria;p__Proteobacteria;c__Deltaproteobacteria;o__Desulfovibrionales;f__Desulfovibrionaceae;g__Bilophila | 0 | 0.000744 | 0.000112 | 0 | 0.000115 | 0.000169 | 0.000284 | 0 | 0.000168 | 0.00028 |
| k__Bacteria;p__Firmicutes;c__Bacilli;o__Lactobacillales;f__Streptococcaceae;g__Streptococcus | 0.000915 | 0.000114 | 0.000168 | 0.00034 | 0.000403 | 0.000281 | 0.000227 | 0.001924 | 0.000225 | 0.000393 |
| k__Bacteria;p__Bacteroidetes;c__Bacteroidia;o__Bacteroidales;f__Rikenellaceae;g__AF12 | 0.000172 | 0 | 0 | 0 | 0 | 0 | 0.000114 | 0.000226 | 0.003931 | 0 |
| k__Bacteria;p__Firmicutes;c__Clostridia;o__Clostridiales;f__Lachnospiraceae;g__Anaerostipes | 0.000172 | 0 | 0 | 0 | 0.000288 | 0 | 0.000114 | 5.66E-05 | 0 | 0 |
| k__Bacteria;p__Firmicutes;c__Bacilli;o__Lactobacillales;f__Leuconostocaceae;g__Unclassified_Leuconostocaceae | 5.72E-05 | 0.001431 | 0 | 0.000113 | 0.00121 | 0.000169 | 0.000511 | 0.002604 | 0.000112 | 0.000112 |
| k__Bacteria;p__Proteobacteria;c__Gammaproteobacteria;o__Vibrionales;f__Vibrionaceae;g__Vibrio | 0 | 0 | 0 | 0 | 0 | 0 | 0 | 0 | 0 | 0 |
| k__Bacteria;p__Firmicutes;c__Clostridia;o__Clostridiales;f__Clostridiaceae;g__Unclassified_Clostridiaceae | 0.000286 | 0.000859 | 0.000112 | 0.000113 | 0.000115 | 0 | 0.000284 | 0.00017 | 0.000899 | 0.000112 |
| k__Bacteria;p__Firmicutes;c__Clostridia;o__Clostridiales;f__Lachnospiraceae;g__Roseburia | 0 | 0 | 0 | 0.000226 | 0 | 0 | 0.00017 | 0 | 0 | 0 |
| k__Bacteria;p__Firmicutes;c__Clostridia;o__Clostridiales;f__Lachnospiraceae;g__Blautia | 0 | 0.000286 | 0.000784 | 0.000113 | 0 | 0 | 0.000227 | 0.000453 | 0.002078 | 0.000112 |
| k__Bacteria;p__Firmicutes;c__Clostridia;o__Clostridiales;f__Ruminococcaceae;g__Faecalibacterium | 5.72E-05 | 0 | 0 | 0 | 0.000173 | 0 | 0.000114 | 0 | 0 | 0 |
| k__Bacteria;p__Bacteroidetes;c__Bacteroidia;o__Bacteroidales;f__[Odoribacteraceae];g__Odoribacter | 0 | 0 | 0 | 0 | 0 | 0 | 0 | 0 | 0 | 0 |
| k__Bacteria;p__Firmicutes;c__Erysipelotrichi;o__Erysipelotrichales;f__Erysipelotrichaceae;g__Coprobacillus | 0 | 0 | 0 | 0 | 0.000692 | 0 | 0.000284 | 0 | 0.001797 | 0.000337 |
| k__Bacteria;p__Firmicutes;c__Clostridia;o__Clostridiales;f__Christensenellaceae;g__Unclassified_Christensenellaceae | 0 | 0.000229 | 5.60E-05 | 0.00017 | 0.000115 | 0 | 0.000284 | 5.66E-05 | 0.000393 | 0.000224 |
| k__Bacteria;p__Proteobacteria;c__Alphaproteobacteria;o__Unclassified_Alphaproteobacteria;f__Unclassified_Alphaproteobacteria;g__Unclassified_Alphaproteobacteria | 0 | 0.000172 | 0.000392 | 0 | 5.76E-05 | 0 | 0 | 0 | 0.001348 | 0 |
| k__Bacteria;p__Firmicutes;c__Clostridia;o__Clostridiales;f__Clostridiaceae;g__Candidatus_Arthromitus | 0 | 0 | 0 | 0 | 0 | 0 | 0 | 0 | 0.000112 | 0.000224 |
| k__Bacteria;p__Firmicutes;c__Clostridia;o__Clostridiales;f__Ruminococcaceae;g__Anaerotruncus | 5.72E-05 | 0.001488 | 5.60E-05 | 0.00017 | 0.000231 | 0 | 0.000341 | 0.000226 | 0 | 0 |
| k__Bacteria;p__Proteobacteria;c__Gammaproteobacteria;o__Pseudomonadales;f__Pseudomonadaceae;g__Unclassified_Pseudomonadaceae | 5.72E-05 | 0.000401 | 0.000784 | 0.00051 | 0 | 0.000169 | 0.000114 | 5.66E-05 | 0.000112 | 0.000168 |
| k__Bacteria;p__Firmicutes;c__Clostridia;o__Clostridiales;f__Lachnospiraceae;g__Pseudobutyrivibrio | 5.72E-05 | 0 | 0 | 0 | 0 | 0.000112 | 0 | 0 | 0 | 0.000673 |
| k__Bacteria;p__Proteobacteria;c__Alphaproteobacteria;o__Rhizobiales;f__Brucellaceae;g__Ochrobactrum | 5.72E-05 | 0 | 5.60E-05 | 5.66E-05 | 0.000173 | 0 | 0.000114 | 0.00017 | 0 | 0.000168 |
| k__Bacteria;p__Firmicutes;c__Erysipelotrichi;o__Erysipelotrichales;f__Erysipelotrichaceae;g__[Eubacterium] | 0 | 0.000458 | 0 | 0 | 0 | 0 | 0 | 0 | 0.001685 | 0 |
| k__Bacteria;p__Firmicutes;c__Bacilli;o__Lactobacillales;f__Unclassified_Lactobacillales;g__Unclassified_Lactobacillales | 0.000114 | 0 | 0.000672 | 0 | 0 | 0 | 0 | 0.000113 | 5.62E-05 | 0.000168 |
| k__Bacteria;p__Tenericutes;c__Mollicutes;o__Anaeroplasmatales;f__Anaeroplasmataceae;g__Anaeroplasma | 0 | 0 | 0 | 5.66E-05 | 0 | 0 | 0.000114 | 0.000113 | 0 | 0 |
| k__Bacteria;p__Firmicutes;c__Bacilli;o__Bacillales;f__Planococcaceae;g__Staphylococcus | 0 | 0 | 0 | 0 | 0 | 0 | 0 | 0.00017 | 0 | 0.00101 |
| k__Bacteria;p__Actinobacteria;c__Coriobacteriia;o__Coriobacteriales;f__Coriobacteriaceae;g__Unclassified_Coriobacteriaceae | 0 | 0 | 0 | 0 | 0.000115 | 0 | 0 | 0 | 0 | 0.000112 |
| k__Bacteria;p__Firmicutes;c__Clostridia;o__Clostridiales;f__Eubacteriaceae;g__Anaerofustis | 0 | 0 | 0.000336 | 5.66E-05 | 5.76E-05 | 0 | 0 | 0 | 0.000393 | 0 |
| k__Bacteria;p__Proteobacteria;c__Alphaproteobacteria;o__RF32;f__Unclassified_RF32;g__Unclassified_RF32 | 0 | 0 | 0 | 0 | 0 | 0 | 0 | 0 | 0.000225 | 0 |
| k__Bacteria;p__Proteobacteria;c__Gammaproteobacteria;o__Pseudomonadales;f__Moraxellaceae;g__Acinetobacter | 0 | 0 | 0 | 0 | 0.000403 | 0 | 0 | 0 | 0 | 0.000112 |
| k__Bacteria;p__Proteobacteria;c__Gammaproteobacteria;o__Enterobacteriales;f__Enterobacteriaceae;g__Proteus | 0 | 0.000114 | 5.60E-05 | 5.66E-05 | 0 | 0 | 0 | 0.000226 | 0 | 0.000112 |
| k__Bacteria;p__Firmicutes;c__Bacilli;o__Lactobacillales;f__Enterococcaceae;g__Enterococcus | 0 | 0 | 0.00028 | 0 | 0 | 0 | 0 | 0.000113 | 0 | 0 |
| k__Bacteria;p__Bacteroidetes;c__Bacteroidia;o__Bacteroidales;f__[Barnesiellaceae];g__Unclassified_[Barnesiellaceae] | 0 | 0.000114 | 0 | 0 | 0 | 0 | 0 | 0 | 0 | 0 |
| k__Bacteria;p__Actinobacteria;c__Actinobacteria;o__Actinomycetales;f__Micrococcaceae;g__Rothia | 0 | 0 | 0 | 0 | 5.76E-05 | 0 | 0 | 0 | 0 | 0 |
| k__Bacteria;p__Firmicutes;c__Clostridia;o__Clostridiales;f__Peptococcaceae;g__Unclassified_Peptococcaceae | 0 | 5.72E-05 | 0 | 0 | 5.76E-05 | 0 | 0 | 0 | 0 | 0 |
| k__Bacteria;p__Firmicutes;c__Bacilli;o__Bacillales;f__Staphylococcaceae;g__Staphylococcus | 0 | 0 | 0 | 0 | 0 | 0 | 0 | 0.000226 | 0 | 0.000112 |
| k__Bacteria;p__Proteobacteria;c__Epsilonproteobacteria;o__Campylobacterales;f__Helicobacteraceae;g__Flexispira | 0 | 0.000114 | 0 | 0.00017 | 0 | 0 | 0 | 0 | 0 | 0 |
| k__Bacteria;p__Proteobacteria;c__Betaproteobacteria;o__Burkholderiales;f__Alcaligenaceae;g__Sutterella | 0 | 0 | 0 | 0 | 0 | 0 | 0 | 0 | 0 | 0 |
| k__Bacteria;p__Cyanobacteria;c__Chloroplast;o__Streptophyta;f__Unclassified_Streptophyta;g__Unclassified_Streptophyta | 0 | 0 | 0 | 0 | 0 | 0 | 0 | 0 | 0 | 0 |
| k__Bacteria;p__Actinobacteria;c__Actinobacteria;o__Actinomycetales;f__Pseudonocardiaceae;g__Amycolatopsis | 0 | 0 | 0 | 0 | 0 | 0 | 0 | 0 | 0 | 0 |
| k__Bacteria;p__Proteobacteria;c__Gammaproteobacteria;o__Xanthomonadales;f__Xanthomonadaceae;g__Unclassified_Xanthomonadaceae | 0 | 0 | 0 | 0 | 5.76E-05 | 0 | 0 | 0 | 0 | 0 |
| k__Bacteria;p__Bacteroidetes;c__[Saprospirae];o__[Saprospirales];f__Chitinophagaceae;g__Sediminibacterium | 0 | 0 | 0 | 0 | 0 | 0 | 0 | 5.66E-05 | 0 | 0 |
| k__Bacteria;p__Firmicutes;c__Erysipelotrichi;o__Erysipelotrichales;f__Erysipelotrichaceae;g__Clostridium | 0 | 0 | 0 | 0 | 5.76E-05 | 0.000112 | 0 | 0 | 0.000112 | 0 |
| k__Bacteria;p__Proteobacteria;c__Betaproteobacteria;o__Neisseriales;f__Neisseriaceae;g__Neisseria | 0 | 0 | 0 | 0 | 0 | 0 | 0 | 0 | 0 | 0 |
| k__Bacteria;p__Proteobacteria;c__Betaproteobacteria;o__Burkholderiales;f__Oxalobacteraceae;g__Cupriavidus | 0 | 0 | 5.60E-05 | 0 | 0 | 0 | 0 | 0 | 0 | 0 |
| No blast hit;Other;Other;Other;Other;Other | 0 | 0 | 0 | 0 | 0 | 0 | 0.000114 | 0.00017 | 0 | 0 |
| k__Bacteria;p__[Thermi];c__Deinococci;o__Thermales;f__Thermaceae;g__Thermus | 0 | 0 | 0 | 5.66E-05 | 0 | 0 | 0 | 5.66E-05 | 0 | 0 |

Table S2- 3 Genus relative abundance of RPGS

| Taxon | RPGS 1 | RPGS 2 | RPGS 3 | RPGS 4 | RPGS 5 | RPGS 6 | RPGS 7 | RPGS 8 | RPGS 9 | RPGS 10 |
| --- | --- | --- | --- | --- | --- | --- | --- | --- | --- | --- |
| k__Bacteria;p__Bacteroidetes;c__Bacteroidia;o__Bacteroidales;f__S24-7;g__Unclassified_S24-7 | 0.415207 | 0.34159 | 0.368722 | 0.541702 | 0.316905 | 0.390049 | 0.411189 | 0.25439 | 0.461328 | 0.417486 |
| k__Bacteria;p__Firmicutes;c__Clostridia;o__Clostridiales;f__Unclassified_Clostridiales;g__Unclassified_Clostridiales | 0.131655 | 0.081845 | 0.035222 | 0.058567 | 0.177925 | 0.124639 | 0.055142 | 0.034001 | 0.154346 | 0.093116 |
| k__Bacteria;p__Bacteroidetes;c__Bacteroidia;o__Bacteroidales;f__Bacteroidaceae;g__Bacteroides | 0.047906 | 0.07511 | 0.067556 | 0.032724 | 0.094695 | 0.182204 | 0.174645 | 0.167934 | 0.00405 | 0.096015 |
| k__Bacteria;p__Verrucomicrobia;c__Verrucomicrobiae;o__Verrucomicrobiales;f__Verrucomicrobiaceae;g__Akkermansia | 0.004582 | 0.0117 | 0.016056 | 0.045254 | 0.130887 | 0.077149 | 0.099118 | 0.041494 | 0.03371 | 0.075607 |
| k__Bacteria;p__Bacteroidetes;c__Bacteroidia;o__Bacteroidales;f__[Paraprevotellaceae];g__[Prevotella] | 0.093899 | 0.10924 | 0.2045 | 0.012754 | 0.005283 | 0.000283 | 0.031837 | 0.160664 | 0.053046 | 0.022909 |
| k__Bacteria;p__Firmicutes;c__Bacilli;o__Lactobacillales;f__Lactobacillaceae;g__Lactobacillus | 0.027491 | 0.06535 | 0.124278 | 0.001287 | 0.002079 | 0.002208 | 0.084574 | 0.045017 | 0.039984 | 0.057473 |
| k__Bacteria;p__Bacteroidetes;c__Bacteroidia;o__Bacteroidales;f__Rikenellaceae;g__Unclassified_Rikenellaceae | 0.075745 | 0.064494 | 0.018833 | 0.085529 | 0.053333 | 0.027396 | 0.021816 | 0.034951 | 0.022416 | 0.024785 |
| k__Bacteria;p__Firmicutes;c__Clostridia;o__Clostridiales;f__Lachnospiraceae;g__Unclassified_Lachnospiraceae | 0.023721 | 0.05462 | 0.034111 | 0.055267 | 0.062212 | 0.044263 | 0.023935 | 0.102617 | 0.02042 | 0.037349 |
| k__Bacteria;p__Bacteroidetes;c__Bacteroidia;o__Bacteroidales;f__Prevotellaceae;g__Prevotella | 0.021111 | 0.045888 | 0.002 | 0.022711 | 0.021131 | 0.042565 | 0.012597 | 0.005536 | 0.020249 | 0.031891 |
| k__Bacteria;p__TM7;c__TM7-3;o__CW040;f__F16;g__Unclassified_F16 | 0.043556 | 0.038354 | 0.002667 | 0.043184 | 0.028268 | 0.027113 | 0.012025 | 0.020412 | 0.043806 | 0.04417 |
| k__Bacteria;p__Firmicutes;c__Clostridia;o__Clostridiales;f__Ruminococcaceae;g__Unclassified_Ruminococcaceae | 0.00928 | 0.008333 | 0.004 | 0.015271 | 0.027313 | 0.009622 | 0.008532 | 0.014428 | 0.006274 | 0.009607 |
| k__Bacteria;p__Firmicutes;c__Clostridia;o__Clostridiales;f__Ruminococcaceae;g__Oscillospira | 0.01131 | 0.00702 | 0.006611 | 0.015774 | 0.016241 | 0.011434 | 0.006814 | 0.011296 | 0.00924 | 0.006651 |
| k__Bacteria;p__Firmicutes;c__Erysipelotrichi;o__Erysipelotrichales;f__Erysipelotrichaceae;g__Unclassified_Erysipelotrichaceae | 0.012064 | 0.01113 | 0.002333 | 0.002797 | 0.004552 | 0.002887 | 0.005841 | 0.004809 | 0.057438 | 0.017907 |
| k__Bacteria;p__Bacteroidetes;c__Bacteroidia;o__Bacteroidales;f__Unclassified_Bacteroidales;g__Unclassified_Bacteroidales | 0.01189 | 0.00508 | 0.001056 | 0.013313 | 0.006688 | 0.005434 | 0.003493 | 0.00302 | 0.002795 | 0.00307 |
| k__Bacteria;p__Proteobacteria;c__Deltaproteobacteria;o__Desulfovibrionales;f__Desulfovibrionaceae;g__Unclassified_Desulfovibrionaceae | 0.008236 | 0.005879 | 0.010389 | 0.013201 | 0.013094 | 0.011943 | 0.014487 | 0.006487 | 0.018538 | 0.002274 |
| k__Bacteria;p__Firmicutes;c__Clostridia;o__Clostridiales;f__Ruminococcaceae;g__Ruminococcus | 0.00812 | 0.005822 | 0.001333 | 0.004307 | 0.015342 | 0.004924 | 0.005039 | 0.002125 | 0.007016 | 0.005003 |
| k__Bacteria;p__Actinobacteria;c__Coriobacteriia;o__Coriobacteriales;f__Coriobacteriaceae;g__Adlercreutzia | 0.013862 | 0.026939 | 0.023889 | 0.009398 | 0.005564 | 0.008434 | 0.009849 | 0.02002 | 0.014545 | 0.021318 |
| k__Bacteria;p__Firmicutes;c__Clostridia;o__Clostridiales;f__Lachnospiraceae;g__[Ruminococcus] | 0.002552 | 0.001883 | 0.038944 | 0.009845 | 0.004271 | 0.010019 | 0.003894 | 0.033274 | 0.003593 | 0.00722 |
| k__Bacteria;p__Bacteroidetes;c__Bacteroidia;o__Bacteroidales;f__Porphyromonadaceae;g__Parabacteroides | 0.002378 | 0.000457 | 0.005111 | 0.000112 | 0.001012 | 0.003283 | 0.00063 | 0.000391 | 0.001027 | 0.001137 |
| k__Bacteria;p__Proteobacteria;c__Gammaproteobacteria;o__Enterobacteriales;f__Enterobacteriaceae;g__Unclassified_Enterobacteriaceae | 0.000348 | 0.000114 | 0 | 0 | 0 | 0 | 0 | 0 | 0.000456 | 0.000171 |
| k__Bacteria;p__Tenericutes;c__Mollicutes;o__RF39;f__Unclassified_RF39;g__Unclassified_RF39 | 0.002494 | 0.007819 | 5.00E-04 | 0.004531 | 0.00118 | 0.000396 | 0.000573 | 0 | 0.002738 | 0.00216 |
| k__Bacteria;p__Bacteroidetes;c__Bacteroidia;o__Bacteroidales;f__Prevotellaceae;g__Unclassified_Prevotellaceae | 0.007366 | 0.007762 | 0.008444 | 0.00151 | 5.62E-05 | 5.66E-05 | 0.002634 | 0.006823 | 0.005191 | 0.002217 |
| k__Bacteria;p__Proteobacteria;c__Deltaproteobacteria;o__Desulfovibrionales;f__Desulfovibrionaceae;g__Desulfovibrio | 0.000348 | 5.71E-05 | 0 | 0 | 0 | 0 | 0 | 0 | 0 | 0.000114 |
| k__Bacteria;p__Firmicutes;c__Clostridia;o__Clostridiales;f__Lachnospiraceae;g__Coprococcus | 0.00145 | 0.001541 | 0.000389 | 0.002797 | 0.002866 | 0.005717 | 0.003264 | 0.000559 | 0.002966 | 0.001251 |
| k__Bacteria;p__Firmicutes;c__Clostridia;o__Clostridiales;f__Dehalobacteriaceae;g__Dehalobacterium | 0.001856 | 0.002397 | 0.006278 | 0.001958 | 0.00281 | 0.00034 | 0.000515 | 0.004138 | 0.000742 | 0.002331 |
| k__Bacteria;p__Firmicutes;c__Clostridia;o__Clostridiales;f__[Mogibacteriaceae];g__Unclassified_[Mogibacteriaceae] | 0.00145 | 0.003025 | 0.001389 | 0.001398 | 0.001236 | 0.002887 | 0.002577 | 0.000951 | 0.00154 | 0.001592 |
| k__Bacteria;p__Proteobacteria;c__Gammaproteobacteria;o__Pseudomonadales;f__Moraxellaceae;g__Unclassified_Moraxellaceae | 0.002146 | 0.004395 | 0.0055 | 0.000503 | 0.000506 | 0.000849 | 0.000458 | 0.004586 | 0.002053 | 0.002501 |
| k__Bacteria;p__Proteobacteria;c__Epsilonproteobacteria;o__Campylobacterales;f__Helicobacteraceae;g__Unclassified_Helicobacteraceae | 0.00087 | 0.000228 | 0.000222 | 0.000727 | 0.000112 | 0 | 0.000458 | 0.012415 | 0.000114 | 0.000171 |
| k__Bacteria;p__Proteobacteria;c__Gammaproteobacteria;o__Pseudomonadales;f__Pseudomonadaceae;g__Pseudomonas | 0.002378 | 0.003139 | 0.005444 | 0.000336 | 0.000169 | 0.000792 | 0.000344 | 0.00302 | 0.001084 | 0.002047 |
| k__Bacteria;p__Firmicutes;c__Clostridia;o__Clostridiales;f__Lachnospiraceae;g__Dorea | 5.80E-05 | 0.000514 | 0.000167 | 0.000112 | 0.00118 | 0.000792 | 0.000229 | 0.000895 | 0.00097 | 0.003581 |
| k__Bacteria;p__Cyanobacteria;c__4C0d-2;o__YS2;f__Unclassified_YS2;g__Unclassified_YS2 | 0 | 0.000114 | 0 | 0 | 0 | 0 | 0 | 0 | 0 | 0 |
| k__Bacteria;p__Proteobacteria;c__Epsilonproteobacteria;o__Campylobacterales;f__Helicobacteraceae;g__Helicobacter | 5.80E-05 | 0 | 0 | 0 | 0 | 0 | 0 | 0.000559 | 0 | 0 |
| k__Bacteria;p__Actinobacteria;c__Actinobacteria;o__Bifidobacteriales;f__Bifidobacteriaceae;g__Bifidobacterium | 0.000232 | 0.000114 | 0 | 0 | 0 | 0 | 0.000344 | 0.000112 | 0.000285 | 0.000114 |
| k__Bacteria;p__Firmicutes;c__Clostridia;o__Clostridiales;f__Clostridiaceae;g__Clostridium | 0.000986 | 0.000114 | 0 | 0.001454 | 0.000843 | 0.000226 | 0.000515 | 5.59E-05 | 0.000171 | 0.001023 |
| k__Bacteria;p__Proteobacteria;c__Deltaproteobacteria;o__Desulfovibrionales;f__Desulfovibrionaceae;g__Bilophila | 0.000696 | 0.000628 | 5.56E-05 | 0.000168 | 0.000731 | 0.000623 | 0.000458 | 0.000839 | 0.000913 | 0.000796 |
| k__Bacteria;p__Firmicutes;c__Bacilli;o__Lactobacillales;f__Streptococcaceae;g__Streptococcus | 0.000348 | 0.001941 | 0.000444 | 5.59E-05 | 0 | 0.000113 | 0 | 0.000671 | 0.000456 | 0.001876 |
| k__Bacteria;p__Bacteroidetes;c__Bacteroidia;o__Bacteroidales;f__Rikenellaceae;g__AF12 | 0 | 0 | 0 | 0 | 0 | 0 | 0 | 0 | 0 | 0 |
| k__Bacteria;p__Firmicutes;c__Clostridia;o__Clostridiales;f__Lachnospiraceae;g__Anaerostipes | 0.00116 | 0.000457 | 0 | 0.000112 | 0 | 0.000226 | 5.73E-05 | 0 | 0.000114 | 0.000227 |
| k__Bacteria;p__Firmicutes;c__Bacilli;o__Lactobacillales;f__Leuconostocaceae;g__Unclassified_Leuconostocaceae | 0 | 0 | 0 | 0 | 0 | 0 | 0 | 0 | 0.002966 | 5.68E-05 |
| k__Bacteria;p__Proteobacteria;c__Gammaproteobacteria;o__Vibrionales;f__Vibrionaceae;g__Vibrio | 0.008932 | 0 | 0 | 0 | 0 | 0 | 0 | 0 | 0 | 0 |
| k__Bacteria;p__Firmicutes;c__Clostridia;o__Clostridiales;f__Clostridiaceae;g__Unclassified_Clostridiaceae | 0.000116 | 0.000571 | 5.56E-05 | 0 | 0.000112 | 0.000226 | 0.000115 | 0 | 0.000342 | 0.000284 |
| k__Bacteria;p__Firmicutes;c__Clostridia;o__Clostridiales;f__Lachnospiraceae;g__Roseburia | 0 | 5.71E-05 | 0 | 0 | 0 | 0 | 0 | 0 | 0 | 0 |
| k__Bacteria;p__Firmicutes;c__Clostridia;o__Clostridiales;f__Lachnospiraceae;g__Blautia | 0 | 0 | 5.56E-05 | 0.000168 | 0.000225 | 0.000226 | 5.73E-05 | 0 | 0 | 0 |
| k__Bacteria;p__Firmicutes;c__Clostridia;o__Clostridiales;f__Ruminococcaceae;g__Faecalibacterium | 0.00029 | 0.0004 | 0.000111 | 5.59E-05 | 0 | 0 | 0 | 0.000559 | 0 | 0.000398 |
| k__Bacteria;p__Bacteroidetes;c__Bacteroidia;o__Bacteroidales;f__[Odoribacteraceae];g__Odoribacter | 0 | 0 | 0 | 0 | 0 | 0 | 0 | 0 | 0 | 0 |
| k__Bacteria;p__Firmicutes;c__Erysipelotrichi;o__Erysipelotrichales;f__Erysipelotrichaceae;g__Coprobacillus | 0.000116 | 0.000457 | 5.56E-05 | 0 | 0 | 0 | 0.000172 | 0 | 0.000342 | 5.68E-05 |
| k__Bacteria;p__Firmicutes;c__Clostridia;o__Clostridiales;f__Christensenellaceae;g__Unclassified_Christensenellaceae | 5.80E-05 | 0.000342 | 5.56E-05 | 0.000224 | 0 | 0.000113 | 0.000286 | 0 | 0 | 0 |
| k__Bacteria;p__Proteobacteria;c__Alphaproteobacteria;o__Unclassified_Alphaproteobacteria;f__Unclassified_Alphaproteobacteria;g__Unclassified_Alphaproteobacteria | 5.80E-05 | 0.000514 | 0 | 0.000168 | 0.000337 | 0.000226 | 0.000687 | 0 | 0.000285 | 0.000227 |
| k__Bacteria;p__Firmicutes;c__Clostridia;o__Clostridiales;f__Clostridiaceae;g__Candidatus_Arthromitus | 0.00029 | 0 | 0.002667 | 0 | 0 | 0 | 5.73E-05 | 0 | 0.000399 | 0 |
| k__Bacteria;p__Firmicutes;c__Clostridia;o__Clostridiales;f__Ruminococcaceae;g__Anaerotruncus | 0 | 0.000114 | 0 | 0 | 0.000337 | 0.000113 | 0 | 0.000112 | 5.70E-05 | 5.68E-05 |
| k__Bacteria;p__Proteobacteria;c__Gammaproteobacteria;o__Pseudomonadales;f__Pseudomonadaceae;g__Unclassified_Pseudomonadaceae | 0.000116 | 0.000228 | 0.000111 | 0.000168 | 5.62E-05 | 0.000113 | 0 | 0.000168 | 5.70E-05 | 5.68E-05 |
| k__Bacteria;p__Firmicutes;c__Clostridia;o__Clostridiales;f__Lachnospiraceae;g__Pseudobutyrivibrio | 0 | 0.000171 | 0 | 0.000392 | 0.00045 | 0 | 0 | 0 | 0.000684 | 0.000398 |
| k__Bacteria;p__Proteobacteria;c__Alphaproteobacteria;o__Rhizobiales;f__Brucellaceae;g__Ochrobactrum | 0.000174 | 0.000856 | 0.000111 | 0.000112 | 0 | 0 | 0 | 0 | 5.70E-05 | 0.000114 |
| k__Bacteria;p__Firmicutes;c__Erysipelotrichi;o__Erysipelotrichales;f__Erysipelotrichaceae;g__[Eubacterium] | 0 | 0 | 0 | 0 | 0 | 0 | 0 | 0 | 0 | 5.68E-05 |
| k__Bacteria;p__Firmicutes;c__Bacilli;o__Lactobacillales;f__Unclassified_Lactobacillales;g__Unclassified_Lactobacillales | 0 | 5.71E-05 | 0 | 0 | 0 | 0 | 0.000401 | 0 | 0.000456 | 0.000341 |
| k__Bacteria;p__Tenericutes;c__Mollicutes;o__Anaeroplasmatales;f__Anaeroplasmataceae;g__Anaeroplasma | 0 | 0.0004 | 0 | 0 | 0 | 0 | 0 | 0 | 5.70E-05 | 0.000512 |
| k__Bacteria;p__Firmicutes;c__Bacilli;o__Bacillales;f__Planococcaceae;g__Staphylococcus | 0.000464 | 0 | 0 | 0 | 0 | 0 | 0 | 0 | 0 | 0 |
| k__Bacteria;p__Actinobacteria;c__Coriobacteriia;o__Coriobacteriales;f__Coriobacteriaceae;g__Unclassified_Coriobacteriaceae | 0.000232 | 0.000114 | 5.56E-05 | 5.59E-05 | 0 | 0 | 0 | 0 | 0.000171 | 0 |
| k__Bacteria;p__Firmicutes;c__Clostridia;o__Clostridiales;f__Eubacteriaceae;g__Anaerofustis | 0 | 5.71E-05 | 0 | 0 | 0 | 0 | 0 | 0 | 0 | 5.68E-05 |
| k__Bacteria;p__Proteobacteria;c__Alphaproteobacteria;o__RF32;f__Unclassified_RF32;g__Unclassified_RF32 | 0 | 0 | 0 | 0 | 0 | 0 | 0 | 0 | 0.000171 | 0 |
| k__Bacteria;p__Proteobacteria;c__Gammaproteobacteria;o__Pseudomonadales;f__Moraxellaceae;g__Acinetobacter | 5.80E-05 | 0 | 0 | 0 | 0 | 0 | 0 | 0.000168 | 0.000114 | 5.68E-05 |
| k__Bacteria;p__Proteobacteria;c__Gammaproteobacteria;o__Enterobacteriales;f__Enterobacteriaceae;g__Proteus | 5.80E-05 | 0 | 0 | 0 | 0 | 0 | 5.73E-05 | 5.59E-05 | 0 | 0 |
| k__Bacteria;p__Firmicutes;c__Bacilli;o__Lactobacillales;f__Enterococcaceae;g__Enterococcus | 0 | 0 | 0 | 0 | 0 | 0 | 0.000286 | 0 | 0.000114 | 0 |
| k__Bacteria;p__Bacteroidetes;c__Bacteroidia;o__Bacteroidales;f__[Barnesiellaceae];g__Unclassified_[Barnesiellaceae] | 5.80E-05 | 0 | 0.000111 | 0 | 0 | 0.000113 | 0 | 0.000112 | 0 | 0.000171 |
| k__Bacteria;p__Actinobacteria;c__Actinobacteria;o__Actinomycetales;f__Micrococcaceae;g__Rothia | 0 | 0 | 0.000111 | 0 | 0 | 0 | 0 | 0 | 0 | 0 |
| k__Bacteria;p__Firmicutes;c__Clostridia;o__Clostridiales;f__Peptococcaceae;g__Unclassified_Peptococcaceae | 0 | 0 | 0 | 0 | 0 | 0 | 0 | 0 | 0 | 0 |
| k__Bacteria;p__Firmicutes;c__Bacilli;o__Bacillales;f__Staphylococcaceae;g__Staphylococcus | 0 | 0 | 0 | 0 | 0 | 0 | 0 | 0 | 5.70E-05 | 0 |
| k__Bacteria;p__Proteobacteria;c__Epsilonproteobacteria;o__Campylobacterales;f__Helicobacteraceae;g__Flexispira | 0 | 0 | 0 | 0 | 0 | 0 | 0 | 0.000168 | 5.70E-05 | 0 |
| k__Bacteria;p__Proteobacteria;c__Betaproteobacteria;o__Burkholderiales;f__Alcaligenaceae;g__Sutterella | 0 | 0 | 0 | 0 | 0 | 0 | 0 | 0.000112 | 0 | 0 |
| k__Bacteria;p__Cyanobacteria;c__Chloroplast;o__Streptophyta;f__Unclassified_Streptophyta;g__Unclassified_Streptophyta | 0.000174 | 0 | 0 | 0 | 0 | 0 | 0 | 0 | 0 | 0 |
| k__Bacteria;p__Actinobacteria;c__Actinobacteria;o__Actinomycetales;f__Pseudonocardiaceae;g__Amycolatopsis | 0 | 0.000228 | 0 | 0 | 0 | 0 | 0 | 0 | 0 | 0 |
| k__Bacteria;p__Proteobacteria;c__Gammaproteobacteria;o__Xanthomonadales;f__Xanthomonadaceae;g__Unclassified_Xanthomonadaceae | 0 | 0.000114 | 0 | 0 | 0 | 0 | 0 | 0 | 0 | 0 |
| k__Bacteria;p__Bacteroidetes;c__[Saprospirae];o__[Saprospirales];f__Chitinophagaceae;g__Sediminibacterium | 0 | 0.000114 | 0 | 0 | 0 | 0 | 0 | 0 | 0 | 0 |
| k__Bacteria;p__Firmicutes;c__Erysipelotrichi;o__Erysipelotrichales;f__Erysipelotrichaceae;g__Clostridium | 0 | 0 | 0 | 0 | 0 | 0 | 0 | 0 | 0 | 0 |
| k__Bacteria;p__Proteobacteria;c__Betaproteobacteria;o__Neisseriales;f__Neisseriaceae;g__Neisseria | 0 | 0 | 5.56E-05 | 0 | 0 | 0 | 0 | 5.59E-05 | 0 | 0 |
| k__Bacteria;p__Proteobacteria;c__Betaproteobacteria;o__Burkholderiales;f__Oxalobacteraceae;g__Cupriavidus | 0 | 0.000114 | 0 | 0 | 0 | 0 | 0 | 0 | 0 | 0 |
| No blast hit;Other;Other;Other;Other;Other | 0 | 0 | 0 | 0 | 0 | 0 | 0 | 0 | 0 | 0 |
| k__Bacteria;p__[Thermi];c__Deinococci;o__Thermales;f__Thermaceae;g__Thermus | 0 | 0 | 0 | 0 | 0 | 0 | 0 | 0 | 0 | 0 |
